# Imidazolium Based Ionic Liquids: Unbiased Recovering of Vaporization Enthalpies from Infinite-Dilution Activity Coefficients

**DOI:** 10.3390/molecules26195873

**Published:** 2021-09-28

**Authors:** Sergey P. Verevkin

**Affiliations:** 1 Department of Physical Chemistry and Department of “Science and Technology of Life, Light and Matter”, University of Rostock, Dr-Lorenz-Weg 2, 18059 Rostock, Germany; sergey.verevkin@uni-rostock.de; 2 Competence Centre CALOR, Faculty of Interdisciplinary Research, University of Rostock, 18059 Rostock, Germany

**Keywords:** ionic liquids, thermodynamics, enthalpy of vaporization, infinite dilution activity coefficient

## Abstract

We propose and test an efficient approach for the assessment of the enthalpies of vaporization of ionic liquids at the reference temperature 298.15 K. The approach is based on activity coefficients at infinite dilution of volatile organic solutes in ionic liquids bearing the imidazolium cation of the general formula [C_n_mim][Anion].

## 1. Introduction

In the last two decades, ionic liquids (ILs) have been considered as potential solvents to replace volatile organic solvents in chemical technologies, as they possess negligible vapour pressure and they cannot evaporate (even at elevated temperatures) and cause air pollution. Typical protic ILs are comprised of a quaternary cation (imidazolium, ammonium, phosphonium, and pyridinium, etc.) with a wide variety of common anions. Their physical and thermodynamic properties can be deliberately tuned by the choice of the cation, anion, and substituents implemented into the cation or anion structure. To develop ILs for the various practical applications, it is important to gain a fundamental knowledge of the factors that control the volatility and thermal stability of pure ILs, as well as the phase behaviour of ionic liquids with other common solvents and chemicals. Since 2001, our laboratory has contributed to this fundamental understanding and prediction of IL properties. We have launched our work with the development of the gas-chromatographic method for determination of infinite-dilution activity coefficients [1]. Activity coefficients at infinite dilution of a solute $\gamma_{1}^{\infty }$ in ionic liquid are important for the optimal design of separation processes. They are especially important when the last traces of impurities must be removed. Knowledge on activity coefficients helps to avoid an oversizing of distillation columns or stripping processes. Moreover, $\gamma_{1}^{\infty }$ values provide information about the intermolecular interactions between solvent and solute; in particular, they are used for the selection of solvents for extraction and extractive distillation.

From the very beginning of the ILs era, there were two crucial advantages propagated for ILs: negligible vapour pressure and inflammability. Both predications have been challenging for our thermochemical lab specialised on vapor pressure measurements and combustion calorimetry. Thus, the long-standing concept of ILs involatility has been disproved with our first quantitative measurements on ILs vapor pressure [2]. Additionally, the acknowledged postulate of ILs inflammability has been disproved by our very first combustion energy measurements of 1-butyl-3-methylimidazolium dicyanamide [3]. Furthermore, we suggested the combination of calorimetric methods (combustion calorimetry and differential scanning calorimetry [4]) with quantum-chemical and molecular dynamic calculations for the “indirect” appraisal of ILs vaporization enthalpies [5]. At the same time, we have intensively developed “direct” experimental methods for ILs vapor pressures and vaporization enthalpy measurements. The main challenges for this method are that, at ambient temperatures, the extremely low vapor pressures of ILs are practically negligible and difficult to measure, whereas, at elevated temperatures, the vaporization process is very often aggravated by the thermal degradation. Nevertheless, we optimized experimental conditions of the conventional transpiration method for the reliable determination of the ILs’ vapor pressures temperature dependences [3,5]. Moreover, we demonstrated that the thermogravimetric analysis (TGA) can be successfully used not only for the common thermal stability studies, but also for the reliable vaporization enthalpy determinations at elevated temperatures [6]. Indeed, it was found that thermal stability of many ILs was not always sufficient to exclude decomposition in the temperature range of the TGA study. In order to overcome the limitations due to the thermal instability of the ILs, we have developed an extremely sensitive quartz-crystal microbalance (QCM) method to determine the ILs’ vapor pressures [7]. The enormous sensitivity of the quartz-crystal (placed over a cavity filled with IL) for the detection of a minute amount of ionic liquid, vaporised and deposited on the surface, has allowed a drastic drop in experimental temperatures (e.g., a starting temperature of the QCM-study of 353 K!) feasible for reliable determination of the mass loss and elimination of thermal decomposition as a consequence. Confronting the ever-questionable thermal stability of ILs at elevated temperatures, we have also invented a method for determining ILs’ volatility at the nanoscale by means of ultra-fast scanning calorimetry (UFSC) [8]. The idea behind this method is to reduce the residence time of the IL sample at all experimental temperatures. It can be achieved by extremely high heating and cooling rates (up to 10^5^ K s^−1^) of the sample placed on the sensor developed for the heat capacity measurements. Thus, the residence time of the IL at high temperature required for measurable mass uptake was optimised to some minima. One consequence was that the chemical processes of thermal degradation hardly have time to begin, as the sample is quickly cooled to ambient temperature. With the UFSC method (based on extremely high heating/cooling rates), much higher experimental temperatures (as compared to common techniques) can be reached without significant decomposition. For example, it was demonstrated that evaporation of 1-ethyl-3-methylimidazolium bis(trifluoromethyl-sulfonyl)imide ([C_2_mim][NTf_2_]) at temperatures of up to 750 K is still the dominating process of mass loss, even at such highly elevated temperatures [8]. Despite the success of the suppression of decomposition during the TGA, QCM, and UFSC measurements, the idea of decreasing the temperatures of IL studies seems to be more attractive and practically relevant. Complementary to the development of the low-temperature QCM-method, we have come up with an idea to use alternating current chip calorimetry (AC) for vapor pressure measurements of ILs at low temperatures [9]. A small droplet of an IL is vaporized isothermally from the chip sensor in a vacuum chamber. The highly sensitive chip sensor (SiN_x_-membrane) allows for mass loss determination at temperatures also starting from 350 K. Finally, it is worth mentioning that the data set on vapor pressures, created for [C_2_mim][NTf_2_] from experiments with AC and UFSC methods for the extremely broad temperature range from 358 K to 780 K, has allowed the estimation of the boiling temperature of this IL. The value (1120 ± 50) K should be considered as the first reliable boiling point of the archetypical ionic liquid obtained from experimental vapor pressures measured in the closest possible proximity to the normal boiling temperature [9].

The systematic development and mutual validation of experimental methods for vapor pressure and vaporisation enthalpies, $\Delta_{l}^{g}H_{m}^{o}$, of the extremely low-volatile ionic liquids delineated above, have been a focus of our scientific interests for the past two decades. As a result, consistent sets of experimental vapor pressures and vaporization enthalpies for imidazolium- [1,2,3,4,5,6,7,8,9,10,11,12,13], pyridinium- [14,15,16,17], pyrrolidinium- [17,18], and phosphonium- [19] based ionic liquids have been reported in the current literature. There is a broad demand for these data, as they are essential for the effective utilization of ILs such as thermofluids, or in separation processes, where precise thermodynamic data on solvents and working fluids are required. Absolute vapor pressures of ILs are indispensable for their modern catalytic applications such as Solid Catalysts with Ionic Liquid Layer (SCILL) [20] or a supported ionic liquid phase (SILP) [21]. The vital question in both cases is: is the IL vapor pressure in the catalytic temperature range low enough in order to avoid the uptake from the catalyst layer? The correct answer is important for the large-scale applications of the generally expensive ionic liquids.

Apart from the practical importance, vaporization enthalpy is one of the key values required for parameterization and optimization of the ILs’ force field for molecular dynamics simulations [22]. Moreover, the systematic information on vaporization energetics within well-defined ionic liquid families opens the way for fundamental understanding of the structure–property relationships in ILs, essential for a reliable prediction of their thermodynamic properties [23]. The latter values are crucial for the optimisation of IL synthesis reactions [24] or IL use for the synthesis of nanoparticles [25].

Admittedly, all standard thermodynamic properties used in practical thermochemical calculations are referenced to any common temperature (most frequently *T* = 298.15 K). As is apparent from the survey of the experimental method for vaporization enthalpy determination outlined above, none of these methods provide the $\Delta_{l}^{g}H_{m}^{o}$(298.15 K) values. As a rule, the measured vaporization enthalpies are referenced to the average temperature *T*_av_ of the experimental interval. These $\Delta_{l}^{g}H_{m}^{o}$(*T*_av_) values have to be adjusted to the reference temperature *T* = 298.15 K according to the Kirchhoff’s Rule [2,3,10] by using appropriate $\Delta_{l}^{g}C_{p,m}^{o}$ values. Formally, the value $\Delta_{l}^{g}C_{p,m}^{o}$ = $C_{p,m}^{o}$(g) − $C_{p,m}^{o}$(l) is the difference of the molar heat capacities of the gaseous $C_{p,m}^{o}$(g) and the liquid phase $C_{p,m}^{o}$(l), respectively. However, the ambiguity and prospects of the $\Delta_{l}^{g}C_{p,m}^{o}$ values required for the adjustment of experimental vaporization enthalpies $\Delta_{l}^{g}H_{m}^{o}$(*T*_av_) to the reference temperature *T* = 298.15 K have already been discussed in detail [10]. Nevertheless, any new idea for independent assessment of $\Delta_{l}^{g}H_{m}^{o}$(298.15 K) values is valuable and desired.

Fortunately, in a series of our recent IL studies [26,27,28] we have been able to show that the $\Delta_{l}^{g}H_{m}^{o}$(298.15 K) values can be derived from the collection of activity coefficients at infinite dilution $\gamma_{1}^{\infty }$ of a solute *1*, in an ionic liquid (solvent *2*) measured by using gas–liquid chromatography (GLC). These studies [26,27,28] were performed on imidazolium based ILs with the trifluoroacetate [CF_3_CO_3_], trifluoromethanesulfonate [CF_3_SO_3_], and methanesulfonate [CH_3_SO_3_] anions. Is this observation also generally valid for imidazolium based ILs containing other types of anions? If yes, the method based on the experimental $\gamma_{1}^{\infty }$ values can be suggested as an independent source for the $\Delta_{l}^{g}H_{m}^{o}$(298.15 K) values, as well as for the valuable validation of $\Delta_{l}^{g}C_{p,m}^{o}$ values applied for the temperature adjustment of experimental $\Delta_{l}^{g}H_{m}^{o}$(*T*_av_) values. Our pioneering paper on GLC determination of activity coefficients of aliphatic and aromatic compounds in 4-methyl-*N*-butyl-pyridinium tetrafluoroborate [1] has inspired the ionic liquid community. Indeed, according to the most recent compilation [29], a comprehensive experimental data base covering 233 ILs and 150 molecular solutes was extracted from 182 references, dealing with activity coefficients at infinite dilution. This huge data pool could be considered as an independent source for the $\Delta_{l}^{g}H_{m}^{o}$(298.15 K) values for numerous differently structured ILs, provided that the procedure for the recovery of vaporisation enthalpies from solute $\gamma_{1}^{\infty }$ values is successful. In order to keep this task in size, in this study we selected only imidazolium-based ILs where the $\Delta_{l}^{g}H_{m}^{o}$(298.15 K) values were measured and evaluated in our recent papers.

## 2. Experimental Background

Activity coefficient at infinite dilution $\gamma_{1}^{\infty }$ of a solute (index 1) in an ionic liquid (index 2) is a valuable thermodynamic tool for the interpretation of intermolecular interactions between a solvent (ILs in this work) and a solute (any volatile compound in this work). Experimental measurements are performed with a commercial gas-chromatograph (GC) equipped with a flame-ionization or a thermal conductivity detector. The ionic liquid (solvent) is used as a stationary phase coating the solid support (diatomaceous earths) in a packed column. Small amounts of organic solutes (0.5 to 2 μL) are injected into the GC column. The retention times are recorded and corrected for the “dead time” (retention time of air or methane).

According to the fundamental work by Cruickshank et al. [30], the retention time is generally proportional to the standardized retention volume $V_{N}$:

(1) $$ln\gamma_{1}^{\infty }=ln\left(\frac{n_{IL}\cdot R\cdot T}{V_{N}\cdot p_{1}^{0}}\right)-\frac{B_{11}-V_{1}^{0}}{RT}\cdot p_{1}^{0}+\frac{2\cdot B_{13}-V_{1}^{\infty }}{RT}\cdot J_{GC}\cdot p_{0}$$

where $p_{1}^{0}$ is the vapour pressure of the pure liquid solute and $n_{IL}$ is the number of moles of the stationary phase component (ionic liquid) on the column. The factor $J_{GC}$ corrects for the influence of the pressure drop along the column. The second and third term in Equation (1) are correction terms which arise from the non-ideality of the mobile gaseous phase. *B*_11_ is the second virial coefficient of the solute, *B*_13_ is the mixed virial coefficient of the solute (1) with the carrier gas nitrogen (index 3), $V_{1}^{0}$ is the liquid molar volume of pure solute, and $V_{1}^{\infty }$ is the partial molar volume of solute in the ionic liquid at infinite dilution (with assumption $V_{1}^{\infty }$ = $V_{1}^{0}$). Molar volumes of solutes $V_{1}^{0}$ are estimated using experimental densities. The values of *B*_11_ and *B*_13_ are estimated according to the Tsonopolous method [31]. A detailed description of the experimental procedure can be found elsewhere [1,30,32]. Values of $\gamma_{1}^{\infty }$ are claimed to be accurate within ± (3 ÷ 5) %. The $\gamma_{1}^{\infty }$ values are usually measured at temperatures not too distant from the reference temperature *T* = 298.15 K and they are practically not affected by the temperature adjustment. For this reason, the $\gamma_{1}^{\infty }$-based estimations of $\Delta_{l}^{g}H_{m}^{o}$(298.15 K) are especially valuable, as they can prove different approaches applied for the temperature adjustments of the vaporization enthalpies from the *T*_av_ to 298.15 K.

## 3. Theoretical Background

In this work we follow the Flory–Huggins theory, which is the main basis of solution and blend thermodynamics [33]. The Flory–Huggins equation handles molecules that are similar chemically, but differ greatly in size. A key value of this theory is a parameter *χ*_12_ quantifying the enthalpic interactions between the components *1* and *2*. The activity coefficient at infinite dilution $\gamma_{1}^{\infty }$ is linked to the Flory−Huggins interaction parameter *χ*_12_ (at infinite dilution) according to the equation [34]:

(2) $$\chi_{12}=\ln\left(\frac{273.15\;\gamma_{1}^{\infty }M_{2}}{TM_{1}}\right)-\left(1-\frac{V_{1}^{*}}{V_{2}^{*}}\right)+\ln\left(\frac{\rho_{1}}{\rho_{2}}\right)$$

where *M*_1_ and *M*_2_ are the molecular weight of solute and solvent, respectively, and *V*_1_^⁎^ and *V*_2_^⁎^ and *ρ*_1_ and *ρ*_2_ are the molar volume and density of solute and solvent, respectively. The Flory–Huggins interaction parameters, *χ*_12_ is related to the Hildebrandt solubility parameters *δ* [34]:

(3) $$\chi_{12}=\frac{V_{1}^{*}{\left(\delta_{1}-\delta_{2}\right)}^{2}}{RT}$$

where *δ*_2_ is the solubility parameter of the IL (solvent) and *δ*_1_ is the solubility parameter of the solute, *R* is the universal gas constant, *T* is the arbitrary temperature, and $V_{1}^{*}$ is the molar volume of the solute at the selected temperature. Solubility parameters are the numerical values that are responsible for the strength of the intermolecular interactions between solute and solvent molecules. The solubility parameters have been widely used as they help to assess the solvation powers of solvents.

The Hildebrand or total solubility parameter (*δ_i_*) is defined as follows [35]:

(4) $$\delta_{i}={\left[(\Delta_{l}^{g}H_{m}^{o}\;-\;RT)/V_{m}\right]}^{0.5}$$

where *V*_m_ is the molar volume, $\Delta_{l}^{g}H_{m}^{o}$ is the standard molar enthalpy of vaporization, *R* is the ideal gas constant, and *T* is the temperature. Vaporization enthalpies, $\Delta_{l}^{g}H_{m}^{o}$, of ionic liquids required for calculations *δ_2_* at *T* = 298.15 K have been systematically evaluated in our recent work. Vaporization enthalpies, $\Delta_{l}^{g}H_{m}^{o}$, of molecular solutes required for calculations *δ_1_* at *T* = 298.15 K were taken from the literature [36,37]. Values of the solubility parameters *δ*_1_ and *δ*_2_ have been calculated according to Equation (4) with help of experimental data on vaporization enthalpies for the solutes [36]. Density values for the solutes were taken from the compilation by Lide [37], and for ILs from the NIST Database [38].

Finally, the algebraic rearrangement of Equation (3) gives:

(5) $$\frac{\delta_{1}^{2}}{RT}-\frac{\chi_{12}}{V_{1}^{*}}=\left(\frac{2\delta_{2}}{RT}\right)\delta_{1}-\frac{\delta_{2}^{2}}{RT}$$

**Figure 1:**
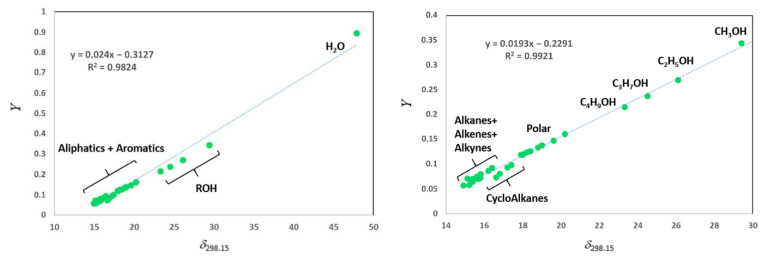
Regression of solubility parameters *δ*_298.15_ of different solutes and *Y*-module (*Y* = (*δ*_1_)^2^/(*RT*) − *χ*_12_/$V_{1}^{*}$ is the left part of Equation (5)) for [C_6_mim][SCN] derived from experimental $\gamma_{1}^{\infty }$ values [39]. (**Left**): the whole data. (**Right**): the same data set, but with the experimental point for water removed.

As it was shown in a typical case (see Figure 1), when the left side of Equation (5) is plotted against *δ*_1_, then the mathematical term 2*δ*_2_/(*RT*) is the slope of the line and the module −$\;\delta_{2}^{2}$/(*RT*) is its intercept. Using linear regression of the experimental data, the slope or intercept can be used to determine *δ*_2_.

We fitted Equation (5) with the solubility parameters *δ*_2_ derived from primary $\gamma_{1}^{\infty }$ values for the [C_n_mim][Anion] available in the literature. For each data set, the solubility parameters *δ*_2_ obtained using the slope and intercept were in agreement with one another within 3%. Thus, the *δ*_2_ values can be estimated as the average from the slope and the intercept. Thus, the vaporization enthalpy, $\Delta_{l}^{g}H_{m}^{o}$(298.15 K), of an IL under study was calculated using the averaged *δ*_2_ value as follows:

(6) $$\Delta_{l}^{g}H_{m}^{o}(T)=[\delta_{2}^{2}\;\times \;V_{m}+RT]$$

where all values, including *V*_m_, are referenced to an arbitrary temperature *T*, which is 298.15 K in this work. Now these $\Delta_{l}^{g}H_{m}^{o}$(298.15 K) results, “indirectly” derived with help of the primary $\gamma_{1}^{\infty }$-values, can be used for comparison with the “direct” experimental results on vaporization enthalpies obtained by the conventional methods.

## 4. Inspection of Activity Coefficients $\gamma_{1}^{\infty }$ Placed at the Disposal

Experimental $\gamma_{1}^{\infty }$ values for different solutes in different types of ILs are regularly appearing in the literature, beginning in 2001 with the aprotic ionic liquids [1] and, ten years later, the protic ionic liquids [40,41]. As a rule, the set of $\gamma_{1}^{\infty }$ values reported for a particular ionic liquid consists of 15 ÷ 40 solutes of different polarity. In the majority of cases, solutes are subdivided into groups: *n*-alkanes with the chain-length C_5_–C_10_, cycloalkanes with the chain-length C_5_–C_8_, alkenes with the chain-length C_5_–C_8_, alkynes with the chain-length C_5_–C_8_, benzene and alkylbenzenes with the chain-length C_1_–C_3_, and linear and branched aliphatic alcohols with the chain-length C_1_–C_5_, as well as a number of solutes of different polarity: acetone, acetonitrile, thiophene, tetrahydrofuran, aliphatic ethers, and esters. In addition, the water is also included if the studies are performed with the thermal conductivity detector. A typical example of the regression of solubility parameters *δ*_298.15_ derived from the experimental $\gamma_{1}^{\infty }$ values is given in Figure 1. For demonstration, we have deliberately chosen the data set containing water (see Figure 1Left). It is apparent that the solubility parameter of water due to the exceedingly high polarity is totally out of correlation with all other types of solutes. Therefore, it is senseless to keep this molecule in the set for regression of solubility parameters. The same data set but without water looks more appropriate (see Figure 1Right), but in this iteration it is obvious that subsets of linear and cyclic aliphatics (alkanes, alkenes, and alkynes) definitely demonstrate individual behavior, and the *δ*_298.15_ points represent a cloud rather than a decent linear attitude. It is noticeable that the cyclical molecules deviate even more drastically from the general trend. We could suggest at least two possible reasons for the variations observed. The first reason is an objective one—the individual behavior of alkane, alkene, and alkyne series can be considered as evidence of specific intermolecular interactions between the different types of aliphatics and the IL under study. Compounds with the double or triple bond are expected to interact with the polar framework of the IL more intensively than the similarly shaped alkanes. Additionally, the cyclic molecules interact with the IL differently than linear molecules, containing the same number of C-atoms. This explanation is in accordance with the general thermodynamic interpretation of the $\gamma_{1}^{\infty }$ values, which are meaningful for interpretation of intermolecular interactions between solutes and solvents.

The second reason for the significant spread of the *δ*_298.15_ points on Figure 1 is a rather subjective one. From our own experiences, the $\gamma_{1}^{\infty }$ measurements are thwarted with considerable technical complications. The main troubles are due to the very short retention times of the highly volatile aliphatics. Even at the low temperatures of the GC experiment, and by using the long packed columns, the retention times of the C_5_–C_7_ hydrocarbons are close to the “dead time”, and the $\gamma_{1}^{\infty }$ values can be significantly affected by the inaccuracy of the time registration. Moreover, in the GC experiment, the C_5_–C_7_ hydrocarbons usually eluate in the immediate vicinity after the solvent peak (e.g., CH_2_Cl_2_). Admittedly, the tail of the solvent peak still contains sufficient residual amounts of solvent molecules dissolved in the IL layer covering the solid support. The solute of interest (one of C_5_–C_7_ hydrocarbons) not only interacts with the IL, but also with the residual CH_2_Cl_2_ molecules and, as a consequence, the true retention time is counterfeit. Thus, the unavoidable solvent peak tailing also contributes to the inaccuracy of the time registration. The practical conclusion for the further $\gamma_{1}^{\infty }$ values acquisition is that the experimental points for C_5_–C_6_ hydrocarbons (alkanes, alkenes, and alkynes) should be omitted.

**Figure 2:**
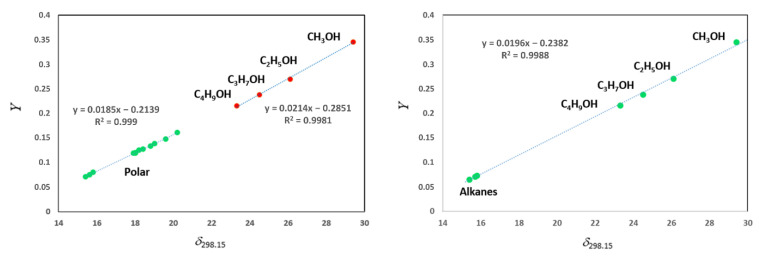
Regression of solubility parameters *δ*_298.15_ of different solutes and *Y*-module (*Y* = (*δ*_1_)^2^/(*RT*) − *χ*_12_/$V_{1}^{*}$ is the left part of Equation (5)) for [C_6_mim][SCN] derived from experimental $\gamma_{1}^{\infty }$ values [39]. (**Left**): separate treatment of collection of polar molecules (see text) and aliphatic alcohols. (**Right**): joint treatment of linear alkanes and linear alcohols.

In contrast to the very volatile C_5_–C_6_ hydrocarbons, the retention times measured for a series of different polar compounds (e.g., acetone, benzene, alkylbenzenes, and ethers) are not affected by the experimental perturbations. Additionally, a reasonable correlation of solubility parameters *δ*_298.15_ of different solutes with the *Y*-module has been virtually observed (see Figure 2Left). However, such a good correlation is rather due to the fact that the series of polar compounds involved in the examination belongs to a relatively narrow range of 0.1 ÷ 0.3 units, according to the normalized solvent polarity scale [42]. For this reason, the moderate intensity of intermolecular interactions between each solute with the IL-solvent is localized at a comparable level, reflecting the individual straight line for the polar compounds in Figure 2Left.

The energetics of interactions of alcohol molecules with the IL is expected to be more profound, as their polarities range from 0.75 (methanol) to 0.60 (1-propanol) in units of the normalized solvent polarity scale [42]. Subsequently, the ROH series (methanol, ethanol, 1-propanol, and 1-butanol) also represents the individual straight line (see Figure 2Left).

## 5. Development of Activity Coefficients $\gamma_{1}^{\infty }$ Data Acquisition and Processing

We have deliberately launched the discussion of the $\gamma_{1}^{\infty }$ measurements with the most consistent data set reported for the for [C_6_mim][SCN] [39]. However, among the available literature compilations on the $\gamma_{1}^{\infty }$ values for imidazolium based ILs of the general formula [C_n_mim][Anion], we have dealt with many less consistent data sets. For example, the $\gamma_{1}^{\infty }$ values for series including benzene and alkylbenzenes, alcohols (1-propanol, 2-propanol, and 2-methyl-1-propanol), polar solutes (acetone, acetonitrile, ethyl acetate, 1,4-dioxane, and tetrahydrofuran), and halogen containing solutes (dichloromethane, trichloromethane, 1,2-dichloromethane, chlorobenzene, and bromobenzene) were reported for [C_6_mim][CF_3_CO_2_] [43].

**Figure 3:**
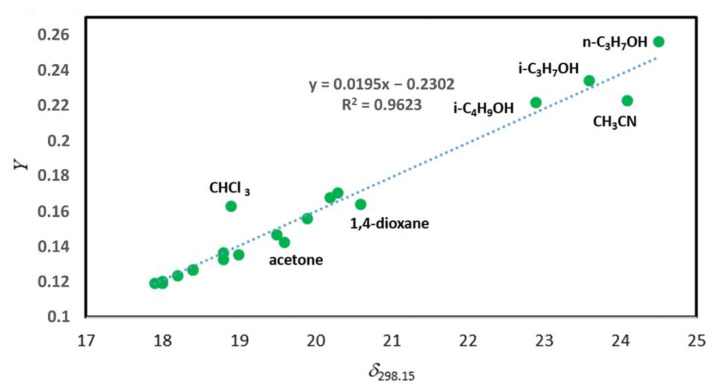
Regression of solubility parameters *δ*_298.15_ of different solutes and *Y*-module (*Y* = (*δ*_1_)^2^/(*RT*) − *χ*_12_/$V_{1}^{*}$ is the left part of Equation (5)) for [C_6_mim][CF_3_CO_2_] derived from experimental $\gamma_{1}^{\infty }$ values [43].

The regression of solubility parameters *δ*_298.15_ of different solutes with the *Y*-module for [C_6_mim][CF_3_CO_2_] is presented in Figure 3. Numerical values used for this correlation are collected in Table S2. The significant spread of the experimental data points is apparent on Figure 3, especially for the polar compounds such as acetone, acetonitrile, 1,4-dioxane, and trichloromethane. Most probably, this scatter is an indication of more pronounced intermolecular interactions between these solutes with the [C_6_mim][CF_3_CO_2_] in comparison to [C_6_mim][SCN]. Certainly, one or the other outlier can be cancelled in order to minimize scattering. However, each removed point inevitably leads step by step to the significant change of the slope and intercept of the regression presented on Figure 3. As a consequence, the *δ_2_*-result (and finally the $\Delta_{l}^{g}H_{m}^{o}$(298.15 K)-result, calculated according to Equation (6)) becomes questionable and virtually dependant on manipulations with the data set on $\gamma_{1}^{\infty }$ values. Moreover, the resulting $\Delta_{l}^{g}H_{m}^{o}$(298.15 K) value for the IL under study becomes crucially dependent on the collection of $\gamma_{1}^{\infty }$ values selected for the evaluation. This conclusion is easy to support with numerical results obtained from Figure 2Right. The value $\Delta_{l}^{g}H_{m}^{o}$(298.15 K) = 115.1 kJ⋅mol^−1^ is calculated using the selection of “polar” solutes. The value $\Delta_{l}^{g}H_{m}^{o}$(298.15 K) = 152.8 kJ⋅mol^−1^ is calculated using only the set of alcohols. Seemingly, such a significant disagreement leaves no hope for practical application of $\gamma_{1}^{\infty }$ values for the $\Delta_{l}^{g}H_{m}^{o}$(298.15 K) appraisal. Nevertheless, let us recall and rationalise the background of the disagreement once more. In general, each available $\gamma_{1}^{\infty }$ data set comprises a series of non-polar solutes (e.g., *n*-alkanes), weakly polar solutes (e.g., alkylbenzenes, ethers, and esters, etc.), and strongly polar solutes (usually alcohols). Each subset exhibits specific intensity of intermolecular interactions with the IL-solvent. Apparently, the slopes of the *δ_1_*(298.15 K)–*Y* regressions (see Figure 1, Figure 2 and Figure 3) constructed for each subset of $\gamma_{1}^{\infty }$-values could be helpful to quantify the specific energetics of these forces. Unfortunately, the scales of the *δ_1_*(298.15 K) values and *Y* values for non-polar and weakly polar solutes are impractically narrow. For example, the variations of *δ_1_*(298.15 K) values for aliphatics are only from 15.2 to 17.4 MPa^0.5^ (see Table S1), and the variations for *Y* values for the same series are from 0.0583 to 0.0989 unities (see Table S1) [26]. As a rule, slopes derived in restricted ranges of parameters are very sensitive and suffer from the individual uncertainties of experimental data points. The variation of *δ_1_*(298.15 K) values and *Y* values for series of weakly polar solutes is somewhat broader (see Table S1), but still insufficient for establishment of robust correlations. Additionally, only the scale of *δ_1_*(298.15 K) values and *Y* values for alcohols ROH with R = methyl, ethyl, *n*-propyl, and *n*-butyl (see Table S1 and Figure 1Right) is adequate for the reliable regression (e.g., the variations of *δ_1_*(298.15 K) values is from 29.4 to 23.3 MPa^0.5^, as well as the variations for *Y* values is from 0.3450 to 0.2155 unities (see Table S1) [26]. It is only regrettable in that papers where at least four C_1_–C_4_ alcohols are measured are quite restricted, precluding any meaningful interpretation of results for this series.

The temporary conclusion for the continuation and development of $\gamma_{1}^{\infty }$ values evaluation is that the none of individual subsets of solutes can be used for a reliable assessment of vaporisation enthalpies $\Delta_{l}^{g}H_{m}^{o}$(298.15 K). However, what speaks against combination of two subsets (see Figure 2Right)? Indeed, by the merging the *n*-alkanes subset with the alcohol subset, we are capturing both edge cases: explicitly weak intermolecular interactions and explicitly strong intermolecular interactions. This idea facilitates an insight into the relationships between activity coefficients $\gamma_{1}^{\infty }$ of solutes in solvent (IL) and vaporisation enthalpy $\Delta_{l}^{g}H_{m}^{o}$(298.15 K) of the solvent (IL). It is the basic knowledge, that activity coefficient $\gamma_{1}^{\infty }$ is the measure for strength of interactions between solute and solvent. Additionally, the vaporization enthalpy is the measure of intermolecular forces in pure solvent. Capturing the $\gamma_{1}^{\infty }$ values only for the edge cases (*n*-alkanes + alcohols), we establish an unbiased average level of intermolecular energetics, which is fixed to the well-defined, restricted set of solutes. Provided that this set will be common for any ionic liquid, the regression of $\gamma_{1}^{\infty }$ values (see Figure 2Right) becomes an unbiased thermodynamic tool for recovering vaporisation enthalpy $\Delta_{l}^{g}H_{m}^{o}$(298.15 K). Such a strict limitation only to the set (*n*-alkanes + alcohols), allows crucial simplification of the $\gamma_{1}^{\infty }$ values evaluation, as the regression *δ_1_*(298.15 K)–*Y* is represented now by practically impeccable straight line (see Figure 2Right). Merging the *n*-alkanes and alcohols subsets is felicitous from the mathematical point of view, as the best possible scales of *δ_1_*(298.15 K) values (from 15.2 to 29.4 MPa^0.5^) and of *Y* values (from 0.0583 to 0.3450 unities) are now encompassed for the $\gamma_{1}^{\infty }$ values evaluation (see Figure 2Right). What is more, the conscious acquisition of only *n*-alkanes and alcohol subsets releases oneself from the troublesome analysis of *δ_1_*(298.15 K)–*Y* regressions (see Figure 1, Figure 2 and Figure 3) and the desperate retrieval of outliers justification. For the sake of brevity, the procedure of $\gamma_{1}^{\infty }$ values evaluation with help of merged alkanes and alcohols subsets along this paper is designate as “heptane–methanol” or *HM* approach (as both solutes are most frequently present in the published data sets and they configure the frame of data acquisition). In our opinion the *HM* approach could be considered as the virtually unbiased tool for examination of $\gamma_{1}^{\infty }$ and $\Delta_{l}^{g}H_{m}^{o}$(298.15 K) interrelations.

## 6. Examination of the *HM* Approach

For a preliminary validation of the *HM* approach it is senseless to examine all 184 available papers [29] on $\gamma_{1}^{\infty }$ values. Imidazolium based ionic liquids have been the most intensively studied in the current literature, and it is reasonable to test *HM* approach with data on the [C_n_mim][Anion] series. Among them, the most popular series of ILs was that associated with the NTf_2_-anion. Enthalpies of vaporisation $\Delta_{l}^{g}H_{m}^{o}$(298.15 K) for this series of ionic liquids have been published recently [10]. They were used for the reconciliation with results derived from $\gamma_{1}^{\infty }$ values reported in the literature [44,45,46,47,48,49,50,51,52,53,54,55]. Most of the data were measured by the GLC technique. Some data sets were also measured by the dilutor technique, but results from both methods have been shown to be indistinguishable. For the sake of transparency, we have preferred the comprehensive studies of activity coefficients, and we have omitted some papers dealing only with separating industrially relevant binary systems (e.g., hexane/hexane, etc.). The $\gamma_{1}^{\infty }$ values available in the literature at temperatures other than *T* = 298.15 K have been adjusted to this reference temperature by using the linear extrapolation ln($\gamma_{1}^{\infty }$) = *f* (1/*T*). We fitted Equation (5) with the solubility parameters *δ*_2_ derived from primary $\gamma_{1}^{\infty }$ values with help of Equations (2)–(4) for the [C_n_mim][Anion] available in the literature, and estimated the $\Delta_{l}^{g}H_{m}^{o}$(298.15 K) values according to Equation (6). Typical results of the data treatment for the [C_n_mim][NTf_2_] series are given in Table 1.

Experimental vaporization enthalpies are listed in column 2. In the first iteration, we processed all $\gamma_{1}^{\infty }$ values presented in the particular paper. The single data point for water was removed from regression, but all other outliers remained at this step. Vaporisation enthalpies calculated in this way are given in column 3. Without going too deeply into the details and peculiarities of each data set, as well as in reason of agreement or disagreement for each individual IL from Table 1, it is quite apparent that the blind application of the traditional *δ_1_*(298.15 K)–*Y* regression is guesswork. Of course, the quality of each individual experimental data point is definitely responsible for the final quality of correlation. However, explicitly for the [C_6_mim][NTf_2_], the special IUPAC project [44] was designed for testing different methods of $\gamma_{1}^{\infty }$ values. According to the final IUPAC conclusions for the majority of measurements where different techniques were used, the agreement is generally within the expected uncertainties for the measurement methods [44]. However, this optimistic conclusion is not able to explain the dramatic scattering of $\Delta_{l}^{g}H_{m}^{o}$(298.15 K) values from 131.4 to 154.0 kJ·mol^−1^ calculated for the [C_6_mim][NTf_2_] (see Table 1, column 3). It is worth mentioning that four contributors [45,46,47,48] of $\gamma_{1}^{\infty }$ values listed for this IL in Table 1 were participants of this IUPAC project. Additionally, what about the *HM* approach? Results derived from this approach are given in Table 1, column 4. Let us examine the *HM* approach applied to [C_6_mim][NTf_2_] data sets, where the $\gamma_{1}^{\infty }$-values are seemingly of certified and impeccable quality. The most comprehensive $\gamma_{1}^{\infty }$-data set was reported by Heintz et al. [45] and it comprised twelve alcohols (from methanol to *n*-hexanol, iso-propyl-, iso-butyl-, sec-butyl-, tert-butyl-, and tert-pentyl-alcohols) and six *n*-alkanes (from *n*-heptane to *n*-dodecane).

The value $\Delta_{l}^{g}H_{m}^{o}$(298.15 K) = 138.5 kJ·mol^−1^ for [C_6_mim][NTf_2_] derived from the $\gamma_{1}^{\infty }$ data set by Heintz et al. [45] is in very good agreement with the experimental value 139.9 ± 1.8 (see Table 1, column 2). The less extensive collection of $\gamma_{1}^{\infty }$ values was reported by Kato and Gmehling [46]. It included only methanol, ethanol, *n*-propanol, iso-propanol, and only two *n*-alkanes (*n*-heptane and *n*-octane). The value $\Delta_{l}^{g}H_{m}^{o}$(298.15 K) = 140.3 kJ·mol^−1^ for [C_6_mim][NTf_2_] derived from their $\gamma_{1}^{\infty }$ data set is also in perfect agreement with the experimental value (see Table 1, column 2). The modest $\gamma_{1}^{\infty }$ data set was reported by Letcher et al. [47] and it consisted from methanol, *n*-heptane, and *n*-octane. The value $\Delta_{l}^{g}H_{m}^{o}$(298.15 K) = 144.4 kJ·mol^−1^ for [C_6_mim][NTf_2_] derived from this very limited $\gamma_{1}^{\infty }$ data set is in acceptable agreement with the experimental value (see Table 1, column 2). The $\gamma_{1}^{\infty }$ data set reported by Dobryakov et al. [48] consists only of eight linear and branched alcohols, thus the data treatment according to the *HP* approach is not possible. However, we could combine the alcohols set from Dobryakov et al. [48] with the reliable *n*-alkanes set from Heintz et al. [45] and the joined treatment of these $\gamma_{1}^{\infty }$ data provides the value $\Delta_{l}^{g}H_{m}^{o}$(298.15 K) = 141.1 kJ·mol^−1^ for [C_6_mim][NTf_2_], which agrees well with the experiment. Summing up the experiences with the *HM* approach application towards [C_6_mim][NTf_2_] data, it is obvious, that the “traditional” treatment (see Table 1, column 3) of the whole set of $\gamma_{1}^{\infty }$ values measured for the non-polar and polar solvent is not able to reproduce the reliable vaporization enthalpies measured by conventional methods (see Table 1, column 2). In contrast, the application of the *HM* approach allows estimated values that can be compared well with the experiment. This conclusion is supported by the comparison of the “theoretical” $\Delta_{l}^{g}H_{m}^{o}$(298.15 K) values derived from the *HM* approach for the [C_n_mim][NTf_2_] series with *n* = 2, 4, 6, 8, 10, and 12 (see Table 1), as well as for the [C_n_mim][CF_3_CO_2_] series with *n* = 2 and 6 (see Table 2).

Such a good correspondence between “theoretical” and experimental $\Delta_{l}^{g}H_{m}^{o}$(298.15 K) values has motivated further systematic comparisons. The results for [C_n_mim][Anion] series with [CF_3_SO_3_], [CH_3_SO_3_], and [Cl] anions are collected in Table 3.

To our surprise, column 2 and column 4 of Table 3 reveal that the “theoretical” values are systematically underestimated in comparison to the “experimental” values. In an attempt to be strict and stringent, we have determined a factor F_im_ = $\Delta_{l}^{g}H_{m}^{o}$(exp)/$\Delta_{l}^{g}H_{m}^{o}$(*HM*) (see Table 1, Table 2, Table 3, Table 4, Table 5 and Table 6 column 5) in order to quantify the degree of underestimation. We found that for the IL series collected in Table 3, the factor F_im_ is close to unity (1.13 to 1.16) for each type of the IL. Additionally, it is important that F_im_ is hardly dependent on the chain-length within each series.

For the development of the *HP* approach it is essential to trace whether the factor F_im_ is accidental, or if it is possibly anion-specific for each [C_n_mim][Anion] series. In Table 4, we evaluated results of regressions for the [C_n_mim][Anion] series with fluorine-containing anions [BF_4_], [PF_6_], and [FAP].

As can be seen from Table 4, the factor F_im_ = 1.28 ± 0.13 for [C_n_mim][BF_4_] and F_im_ = 1.41 ± 0.03 for [C_n_mim][PF_6_] are closely within their combined uncertainties. Additionally, the chain-length dependence of the F_im_ factors is absent in both series. Moreover, the increase in the number of F atoms in the anion from 4 in [BF_4_] to 6 in [PF_6_] does not seem to increase the F_im_ factor. In order to reinforce this conclusion, we extended our study with the perfluorinated anion [FAP]. Astonishingly, the factor F_im_ = 0.94 ± 0.02 (see Table 4 for [C_n_mim][FAP]) does not fulfil our expectations. Hence, it is clear that the factor F_im_ is virtually anion-dependent. However, F_im_ does seem to be chain-length independent (it is explicitly verified in Table 4, column 5). These two conclusions have been additionally proven with the available $\gamma_{1}^{\infty }$ data sets for [C_n_mim][NO_3_] (see F_im_= 1.44 ± 0.06 in Table 5), as well as with the data sets for cyano-containing ionic liquids [C_n_mim][Anion] with [SCN], [DCA], [TCM], and [TCB] anions (see Table 6). As shown in this table, the increase in the number of CN-groups from one in thiocyanate, to two in di-cyano-amide, and three in tri-cyano-methane, does not seem to increase the F_im_ factor (F_im_ ≈ 1.3 to 1.4 within the combined experimental uncertainties). However, an accumulation of four CN-groups in the [TCB] reduces the factor significantly to the value of F_im_ = 1.19 ± 0.08 (see Table 6 for [C_n_mim][TCB]). Such a trend is similar to the accumulation of F atoms in the [C_n_mim] [FAP] series (see Table 4) and should be investigated more closely.

As a final conclusion, it has now been proven that the *HM* approach can generally be used to estimate of $\Delta_{l}^{g}H_{m}^{o}$(298.15 K) of [C_n_mim][Anion] by using the anion specific correction factors F_im_ to reconcile “theoretical” and “experimental” values. The variation of the F_im_ factors in the narrow range from 1 to 1.4 localized in this work enables a quick assessment of the general $\Delta_{l}^{g}H_{m}^{o}$(298.15 K) level, even with the averaged factor F_im_ = 1.2. However, these rough estimates could be aggravated by the uncertainties of 5–6 kJ·mol^−1^, provided that good quality $\gamma_{1}^{\infty }$ data were used in the evaluation. Nevertheless, such agreement between “conventional” $\Delta_{l}^{g}H_{m}^{o}$(298.15 K) values and those “theoretical” from the *HM* approach can be considered as acceptable, taking into account the experimental uncertainties at the level 2 to 3 kJ·mol^−1^. The application of the anion-specific F_im_ factors (see Table 1, Table 2, Table 3, Table 4, Table 5 and Table 6) can improve agreement between “theoretical” and “experimental” vaporization enthalpies to 2–4 kJ·mol^−1^. Taking into account the difficulties described in the introduction when measuring the enthalpies of vaporization of the extremely low-volatile ionic liquids, the *HM* approach is considered a valuable indirect complementary method. It opens a wide window of opportunity to collect a large amount of reliable data for studies on structure–property relationships for ionic liquids. Moreover, the $\Delta_{l}^{g}H_{m}^{o}$(298.15 K) values derived from the *HM* approach could help to resolve contradictions with the adjustments of vaporization enthalpies from experimental elevated temperatures to the reference temperature *T* = 298.15 K.

During this study, we have frequently noticed that methanol on the regression plot [δ_1_(298.15 K)—Y] was slightly above the general trend (e.g., Figure 1Right and Figure 2Right). At the same time, the experimental point of ethanol met the line better. We use this observation in order to modify the (Heptane-Methanol) approach to the (Heptane-Ethanol) approach (or *HE* approach). To do this, we excluded methanol from the primary $\gamma_{1}^{\infty }$ data sets and recalculated the anion-specific F_im_ factors (see Tables S2–S8). The *HE* approach with the specific F_im_ factors can be helpful in the evaluation of the primary $\gamma_{1}^{\infty }$ data sets, for which methanol was not included or the activity coefficient of methanol is of questionable quality. The reasonable combination of the *HM* and *HE* approaches increases the unbiased flexibility of the $\gamma_{1}^{\infty }$ data sets evaluation and estimation of reliable $\Delta_{l}^{g}H_{m}^{o}$(298.15 K) values.

## 7. Outlook for the *HM* Approach

Evaluation of available $\gamma_{1}^{\infty }$ data sets for [C_n_mim][Anion] series of ionic liquids has generally demonstrated the applicability of the *HM* approach for the independent appraisal of $\Delta_{l}^{g}H_{m}^{o}$(298.15 K) values. However, there are at least two open questions which should be studied in the future:

— are the correction factors F_im_ designed in this work for [C_n_mim][Anion] series common for the pyridinium, pyrrolidinium, ammonium, amd phosphonium, etc. based ionic liquids connected to the same anion?
— is the peculiarity observed for the factor F_im_ of the poly-fluorinated and poly-cyano substituted anions connected to the imidazolium cation common for the ionic liquids with other cations? It is easy, if time consuming, to complete this task, as there is already a wealth of $\gamma_{1}^{\infty }$ literature; it will be examined in the forthcoming work. Nonetheless, in critically working with the large amount of primary $\gamma_{1}^{\infty }$ data and having significant experiences with the GLC method, we have generated few essential recommendations for the future experimental work; in the case if the $\gamma_{1}^{\infty }$-values will be deliberately measured for $\Delta_{l}^{g}H_{m}^{o}$(298.15 K) estimations:

subset of *n*-alkanes should encompass solutes at least from *n*-heptane to *n*-dodecane.

subset of *n*-alcohols should include solutes at least from methanol to *n*-hexanol.

the amount of ionic liquid should be about 35 ÷ 45 mass percent of the support material.

retention times are advisable to measure with the help of two columns of different length (e.g., 50 cm and 100 cm). This advice helps to obtain sharp peaks for both low and high volatility representatives of *n*-alkanes and especially for *n*-alcohol subgroups. The temperature range of the GLC experiment should be as close as possible to the reference temperature *T* = 298.15 K. The column temperature has to be low enough to escape the solvent tailing (see Figure S1b) for small alkanes. However, the column temperature has to be high enough to avoid the solute peak broadening (see Figure S1c), leading to random errors of time measurements. Implementation of two columns of different length helps to keep the experimental temperature range in optimum.

In our opinion, following these recommendations, a complementary method for reliable assessment of $\Delta_{l}^{g}H_{m}^{o}$(298.15 K) values will be established.

## Figures and Tables

**Table 1:** [C_n_mim][NTf_2_] series: comparison of $\Delta_{l}^{g}H_{m}^{o}$ (298.15 K) values derived according to Equations (5) and (6) with experimental results from conventional methods.

IL	$\Delta_{l}^{g}H_{m}^{o}(\exp){\;}^{a}$	$\Delta_{l}^{g}H_{m}^{o}(\mathrm{GLC}){\;}^{b}$	$\Delta_{l}^{g}H_{m}^{o}(\mathrm{HM}){\;}^{c}$	F_im_^d^	Ref. ^e^
[C_2_mim][NTf_2_]	127.7 ± 1.8 [10]	119.0	125.3	1.02	[49]
		115.3	125.5	1.02	[50]
		133.3	134.1	0.95	[51]
[C_4_mim][NTf_2_]	134.9 ± 1.7 [10]	129.2	134.3	1.00	[52]
			132.7	1.02	[53]
[C_6_mim][NTf_2_]	139.9 ± 1.8 [10]	133.9	138.5	1.01	[45]
		131.4	140.3	1.00	[46]
		143.5	144.4	0.97	[47]
			141.1 ^f^	0.99	[45,48]
[C_8_mim][NTf_2_]	147.1 ± 2.0 [10]	145.8	148.4	0.99	[46]
[C_10_mim][NTf_2_]	154.6 ± 2.5 [10]	151.1	155.0	1.00	[54]
[C_12_mim][NTf_2_]	161.5 ± 1.8 [10]		161.1	1.00	[55]
				1.00 ± 0.01	

^a^ Experimental values derived from different conventional techniques and evaluated and reported in our recent papers. ^b^ Estimated according to Equations (5) and (6) using $\gamma_{1}^{\infty }$ values for all solutes reported in original papers. ^c^ Estimated according to Equations (5) and (6) using $\gamma_{1}^{\infty }$ values for only *n*-alkanes and alcohols reported in original papers. ^d^ Correction factor F_im_ = $\Delta_{l}^{g}H_{m}^{o}$(exp)/$\Delta_{l}^{g}H_{m}^{o}$(*HM*). ^e^ References for the experimental $\gamma_{1}^{\infty }$ values. ^f^ Combination of $\gamma_{1}^{\infty }$ data set of alcohols [48] with the $\gamma_{1}^{\infty }$ data set for *n*-alkanes [45].

**Table 2:** [C_n_mim][CF_3_CO_2_] series: comparison of $\Delta_{l}^{g}H_{m}^{o}$ (298.15 K) values derived according to Equations (5) and (6) with experimental results from conventional methods ^a^.

IL	$\Delta_{l}^{g}H_{m}^{o}(\exp)$	$\Delta_{l}^{g}H_{m}^{o}(\mathrm{GLC})$	$\Delta_{l}^{g}H_{m}^{o}(\mathrm{HM})$	F_im_	Ref.
[C_2_mim][CF_3_CO_2_]	126.4 ± 1.5 [26]	122.3	126.4	1.00	[56]
[C_6_mim][CF_3_CO_2_]	141.5 ± 1.9 [26]	183.2	143.8	0.98	[43]
				0.99 ± 0.01	

^a^ The definition of symbols is the same as in Table 1.

**Table 3:** [C_n_mim][Anion] series with [CF_3_SO_3_], [CH_3_SO_3_], and [Cl]: comparison of $\Delta_{l}^{g}H_{m}^{o}$ (298.15 K) values derived according to Equations (5) and (6) with experimental results from conventional methods ^a^.

IL	$\Delta_{l}^{g}H_{m}^{o}(\exp)$	$\Delta_{l}^{g}H_{m}^{o}(\mathrm{GLC})$	$\Delta_{l}^{g}H_{m}^{o}(\mathrm{HM})$	F_im_	Ref.
[C_2_mim][CF_3_SO_3_]	134.2 ± 2.5 [27]	108.0	110.1	1.22	[57]
[C_4_mim][CF_3_SO_3_]	138.7 ± 2.4 [27]	116.7 ^b^	120.6	1.15	[58,59]
		121.2	123.9	1.12	[60]
[C_6_mim][CF_3_SO_3_]	146.1 ± 2.4 [27]	137.6 ^c^			[61]
				1.16 ± 0.03	
[C_2_mim][CH_3_SO_3_]	141.4 ± 1.9 [28]	120.5	120.0	1.18	[62]
		111.8	121.0	1.17	[63]
		122.7	123.1	1.15	[64]
[C_4_mim][CH_3_SO_3_]	149.8 ± 2.1 [28]	126.5	136.6	1.10	[65]
				1.15 ± 0.02	
[C_4_mim][Cl]	153.3 ± 1.9 [66]	140.5	138.3	1.11	[65]
[C_6_mim][Cl]	160.5 ± 2.4 [66]	132.2	135.0	1.19	[67]
[C_8_mim][Cl]	166.8 ± 3.8 [66]		154.9	1.08	[65]
				1.13 ± 0.03	

^a^ The definition of symbols is the same as in Table 1. ^b^ Combination of $\gamma_{1}^{\infty }$ data set of *n*-alkanes [58] with the $\gamma_{1}^{\infty }$ data set for alcohols [59]. ^c^ Estimated from data set containing $\gamma_{1}^{\infty }$ values only for alkylbenzenes and low polar solutes.

**Table 4:** [C_n_mim][Anion] series with fluorine-containing anions: comparison of $\Delta_{l}^{g}H_{m}^{o}$ (298.15 K) values derived according to Equations (5) and (6) with experimental results from conventional methods ^a^.

IL	$\Delta_{l}^{g}H_{m}^{o}(\exp)$	$\Delta_{l}^{g}H_{m}^{o}(\mathrm{GLC})$	$\Delta_{l}^{g}H_{m}^{o}(\mathrm{HM})$	F_im_	Ref.
[C_2_mim][BF_4_]	130.5 ± 2.9 [16]	101.1	103.2	1.26	[68]
		95.3	104.1	1.25 ^b^	[69]
[C_4_mim][BF_4_]	138.3 ± 2.7 [16]	106.5	108.1	1.28	[70]
		105.0	108.5	1.27	[71]
		96.4	102.7	1.35	[68]
		99.3	103.2 ^c^	1.34	[72,73]
			98.4 ^c^	1.41	[71,74]
			103.1 ^c^	1.34	[71,75]
[C_6_mim][BF_4_]	146.8 ± 2.7 [16]	118.4	118.5	1.24	[76]
		107.9	113.3	1.30	[68]
			112.0 ^c^	1.31	[68,75]
[C_8_mim][BF_4_]	152.8 ± 2.8 [16]	117.0	118.8	1.29	[77]
		116.7	119.8	1.28	[68]
		142.9	142.3	1.07	[70]
[C_10_mim][BF_4_]	158.3 ± 2.8 [16]	132.4			[78]
[C_12_mim][BF_4_]	167.3 ± 2.9		132.9	1.26 ^e^	[79]
			131.0	1.28 ^f^	[79]
[C_14_mim][BF_4_]	179.4 ± 3.1		148.5	1.21 ^e^	[79]
			137.9	1.30 ^f^	[79]
[C_16_mim][BF_4_]	180.5 ± 3.5 ^d^	143.6	142.7	1.26	[80]
				1.28 ± 0.13	
[C_4_mim][PF_6_]	146.5 ± 2.6 [13]	96.0	101.3	1.45	[81]
		75.4	96.7 ^c^	1.51 ^c^	[81,82]
		124.5	99.1 ^c^	1.48 ^c^	[81,83]
			99.9	1.47	[84]
			97.5 ^c^	1.50 ^c^	[48,81]
[C_6_mim][PF_6_]	150.8 ± 2.7 [13]	111.0	111.5	1.35	[85]
		85.7			[86]
[C_8_mim][PF_6_]	154.9 ± 2.8 [13]	128.7	111.1	1.39 ^c^	[86,87]
		128.5	128.4	1.21	[87]
		115.0	115.4	1.34	[88]
				1.41 ± 0.03	
[C_2_mim][FAP] ^g^	126.4 ± 1.9 [89]	130.1	135.4	0.93	[90]
		123.3	129.6	0.98	[91]
[C_6_mim][FAP] ^g^	141.5 ± 1.9 [89]	158.0	156.0	0.91	[62]
				0.94 ± 0.02	

^a^ The definition of symbols is the same as in Table 1. ^b^ Experimental data on alcohols were absent in this data set. ^c^ Combination of $\gamma_{1}^{\infty }$ data set of alcohols with the $\gamma_{1}^{\infty }$ data set for *n*-alkanes reported in different papers, as given in the last column. ^d^ Extrapolated value based on experimental data [16]. ^e^ Activity coefficients were measure in the liquid phase. ^f^ Activity coefficients were measured in the liquid-crystal phase. ^g^ [FAP] = tris(pentafluoroethyl)trifluorophosphate anion.

**Table 5:** [C_n_mim][NO_3_] series: comparison of $\Delta_{l}^{g}H_{m}^{o}$ (298.15 K) values derived according to Equations (5) and (6) with experimental results from conventional methods ^a^.

IL	$\Delta_{l}^{g}H_{m}^{o}(\exp)$	$\Delta_{l}^{g}H_{m}^{o}(\mathrm{GLC})$	$\Delta_{l}^{g}H_{m}^{o}(\mathrm{HM})$	F_im_	Ref.
[C_2_mim][NO_3_]	158.1 ± 5.3 [92]	90.0	96.7	1.63	[93]
[C_4_mim][NO_3_]	162.3 ± 5.3 [92]	109.5	115.2	1.41	[94]
[C_4_mim][NO_3_]	162.3 ± 5.3 [92]	112.3	111.8	1.45	[95]
[C_6_mim][NO_3_]	170.1 ± 5.3 [92]	121.6	120.4 ^b^	1.41	[96]
[C_8_mim][NO_3_]	177.9 ± 5.3 [92]	136.5	138.9	1.28	[97]
				1.44 ± 0.06	

^a^ The definition of symbols is the same as in Table 1. ^b^ The $\gamma_{1}^{\infty }$ data set for *n*-alkanes were not reported in the original work; they were assessed in this work according to the trend with the chain-length.

**Table 6:** [C_n_mim][Anion] series with cyano-containing anions: comparison of $\Delta_{l}^{g}H_{m}^{o}$ (298.15 K) values derived according to Equations (5) and (6) with experimental results from conventional methods ^a^.

IL	$\Delta_{l}^{g}H_{m}^{o}(\exp)$	$\Delta_{l}^{g}H_{m}^{o}(\mathrm{GLC})$	$\Delta_{l}^{g}H_{m}^{o}(\mathrm{HM})$	F_im_	Ref.
[C_2_mim][SCN]	153.6 ± 1.8 [98,99]	100.9	101.2	1.52	[100]
[C_4_mim][SCN]	156.9 ± 2.5 [98]	116.4	119.9	1.31	[101]
[C_6_mim][SCN]	164.7 ± 3.0 [98]	122.8	124.4	1.32	[39]
				1.38 ± 0.07	
[C_2_mim][DCA]	155.6 ± 3.1 [3,5]	102.8	105.2	1.48	[102]
[C_4_mim][DCA]	162.9 ± 3.4 [3,5]	113.9	122.0	1.34	[103]
				1.41 ± 0.07	
[C_2_mim][TCM]	140.3 ± 3.0 [5,104]	100.8	106.1	1.32	[105]
[C_4_mim][TCM]	146.9 ± 3.0 [5,104]	117.7	120.2	1.22	[106]
				1.27 ± 0.05	
[C_2_mim][TCB]	130.1 ± 2.5 [107]	98.1	100.4	1.30	[108]
		94.4	97.2	1.34	[109]
[C_6_mim][TCB]	143.0 ± 2.3 [107]	120.8	124.4	1.15	[110]
[C_10_mim][TCB]	157.1 ± 2.5 [107]	155.1	157.9	0.99	[111]
				1.19 ± 0.08	

^a^ The definition of symbols is the same as in Table 1. [SCN] = thiocyanate; [DCA] = di-cyanoamide; [TCM] = tri-cyano-methane; and [TCB] = tetra-cyano-borate.

## Data Availability

Not applicable.

## Supplementary Materials

The following are available online, Figure S1: Typical gas-chromatographic charts from $\gamma_{1}^{\infty }$-measurements, Table S1: Data used for regression of [C_6_mim][SCN] solubility parameters, Table S2: Data used for regression of [C_6_mim][CF_3_CO_2_] solubility parameters, Table S3: Comparison of vaporization enthalpies derived from activity coefficients with experimental results from conventional methods for [C_n_mim][NTf_2_] series. Table S4: Comparison of vaporization enthalpies derived from activity coefficients with experimental results from conventional methods for [C_n_mim][NTf_2_] series. Table S3: Comparison of vaporization enthalpies derived from activity coefficients with experimental results from conventional methods for [C_n_mim][NTf_2_] series. Table S3: Comparison of vaporization enthalpies derived from activity coefficients with experimental results from conventional methods for [C_n_mim][NTf_2_] series. Table S4: Comparison of vaporization enthalpies derived from activity coefficients with experimental results from conventional methods for [C_n_mim][CF_3_CO_2_] series. Table S5: Comparison of vaporization enthalpies derived from activity coefficients with experimental results from conventional methods for [C_n_mim][Anion] series with [CF_3_SO_3_], [CH_3_SO_3_], and [Cl] anions, Table S6: Comparison of vaporization enthalpies derived from activity coefficients with experimental results from conventional methods for [C_n_mim][Anion] series with fluorine containing anions, Table S7: Comparison of vaporization enthalpies derived from activity coefficients with experimental results from conventional methods for [C_n_mim][NO_3_] series, Table S8: Comparison of vaporization enthalpies derived from activity coefficients with experimental results from conventional methods for [C_n_mim][Anion] series with cyano-containing anions.

## References

[1] Heintz A., Kulikov D.V., Verevkin S.P. (2001). Thermodynamic Properties of Mixtures Containing Ionic Liquids. 1. Activity Coefficients at Infinite Dilution of Alkanes, Alkenes, and Alkylbenzenes in 4-Methyl-n-butylpyridinium Tetrafluoroborate Using Gas−Liquid Chromatography. J. Chem. Eng. Data.

[2] Zaitsau D.H., Kabo G.J., Strechan A.A., Paulechka Y.U., Tschersich A., Verevkin S.P., Heintz A. (2006). Experimental Vapor Pressures of 1-Alkyl-3-methylimidazolium Bis(trifluoromethylsulfonyl)imides and a Correlation Scheme for Estimation of Vaporization Enthalpies of Ionic Liquids. J. Phys. Chem. A.

[3] Emel’yanenko V.N., Verevkin S.P., Heintz A. (2007). The Gaseous Enthalpy of Formation of the Ionic Liquid 1-Butyl-3-methylimidazolium Dicyanamide from Combustion Calorimetry, Vapor Pressure Measurements, and Ab Initio Calculations. J. Amer. Chem. Soc..

[4] Verevkin S.P., Zaitsau D.H., Emel’yanenko V.N., Schick C., Jayaraman S., Maginn E.J. (2012). An elegant access to formation and vaporization enthalpies of ionic liquids by indirect DSC experiment and “in silico” calculations. Chem. Comm..

[5] Verevkin S.P., Emel’yanenko V.N., Zaitsau D.H., Heintz A., Muzny C.D., Frenkel M.L. (2010). Thermochemistry of imidazolium-based ionic liquids: Experiment and first-principles calculations. Phys. Chem. Chem. Phys..

[6] Verevkin S.P., Ralys R.V., Zaitsau D.H., Emel’yanenko V.N., Schick C. (2012). Express thermo-gravimetric method for the vaporization enthalpies appraisal for very low volatile molecular and ionic compounds. Thermochim. Acta.

[7] Verevkin S.P., Zaitsau D.H., Emel’yanenko V.N., Heintz A. (2011). A New Method for the Determination of Vaporization Enthalpies of Ionic Liquids at Low Temperatures. J. Phys. Chem. B.

[8] Ahrenberg M., Brinckmann M., Schmelzer J.W.P., Beck M., Schmidt C., Keßler O., Kragl U., Verevkin S.P., Schick C. (2014). Determination of volatility of ionic liquids at the nanoscale by means of ultra-fast scanning calorimetry. Phys. Chem. Chem. Phys..

[9] Ahrenberg M., Beck M., Schmidt C., Keßler O., Kragl U., Verevkin S.P., Schick C. (2016). Vapor pressure of ionic liquids at low temperatures from AC-chip-calorimetry. Phys. Chem. Chem. Phys..

[10] Verevkin S.P., Zaitsau D.H., Emel’yanenko V.N., Yermalayeu A.V., Schick C., Liu H., Maginn E.J., Bulut S., Krossing I., Kalb R. (2013). Making Sense of Enthalpy of Vaporization Trends for Ionic Liquids: New Experimental and Simulation Data Show a Simple Linear Relationship and Help Reconcile Previous Data. J. Phys. Chem. B.

[11] Zaitsau D.H., Yermalayeu A.V., Verevkin S.P., Bara J.E., Stanton A.D. (2013). Structure–Property Relationships in Ionic Liquids: A Study of the Influence of N(1) Ether and C(2) Methyl Substituents on the Vaporization Enthalpies of Imidazolium-Based Ionic Liquids. Ind. Eng. Chem. Res..

[12] Zaitsau D.H., Yermalayeu A.V., Verevkin S.P., Bara J.E., Wallace D.A. (2015). Structure-property relationships in ionic liquids: Chain length dependence of the vaporization enthalpies of imidazolium-based ionic liquids with fluorinated substituents. Thermochim. Acta.

[13] Zaitsau D.H., Yermalayeu A.V., Emel’yanenko V.N., Butler S., Schubert T., Verevkin S.P. (2016). Thermodynamics of Imidazolium-Based Ionic Liquids Containing PF6 Anions. J. Phys. Chem. B.

[14] Emel’yanenko V.N., Verevkin S.P., Heintz A. (2011). Pyridinium based ionic liquids. N-Butyl-3-methyl-pyridinium dicyanoamide: Thermochemical measurement and first-principles calculations. Thermochim. Acta.

[15] Zaitsau D.H., Yermalayeu A.V., Emel’yanenko V.N., Verevkin S.P., Welz-Biermann U., Schubert T. (2012). Structure-property relationships in ILs: A study of the alkyl chain length dependence in vaporisation enthalpies of pyridinium based ionic liquids. Sci. China Chem..

[16] Zaitsau D.H., Yermalayeu A.V., Emel’yanenko V.N., Schulz A., Verevkin S.P. (2017). Thermochemistry of Pyridinium Based Ionic Liquids with Tetrafluoroborate Anion. Z. Anorg. Allg. Chem..

[17] Verevkin S.P., Ralys R.V., Emel’yanenko V.N., Zaitsau D.H., Schick C. (2013). Thermochemistry of the pyridinium- and pyrrolidinium-based ionic liquids. J. Therm. Anal. Calorim..

[18] Emel’yanenko V.N., Verevkin S.P., Heintz A., Corfield J.-A., Deyko A., Lovelock K.R.J., Licence P., Jones R.G. (2008). Pyrrolidinium-Based Ionic Liquids. 1-Butyl-1-methyl Pyrrolidinium Dicyanoamide: Thermochemical Measurement, Mass Spectrometry, and ab Initio Calculations. J. Phys. Chem. B.

[19] Zaitsau D.H., Plechkova N., Verevkin S.P. (2019). Vaporization thermodynamics of ionic liquids with tetraalkylphosphonium cations. J. Chem. Thermodyn..

[20] Kernchen U., Etzold B., Korth W., Jess A. (2007). Solid Catalyst with Ionic Liquid Layer (SCILL)—A New Concept to Improve Selectivity Illustrated by Hydrogenation of Cyclooctadiene. Chem. Eng. Technol..

[21] Fehrmann R., Riisager A., Haumann M. (2014). Supported Ionic Liquids: Fundamentals and Applications.

[22] Bedrov D., Piquemal J.-P., Borodin O., MacKerell A.D., Roux B., Schröder C. (2019). Molecular Dynamics Simulations of Ionic Liquids and Electrolytes Using Polarizable Force Fields. Chem. Rev..

[23] Verevkin S.P. (2008). Predicting Enthalpy of Vaporization of Ionic Liquids: A Simple Rule for a Complex Property. Angew. Chem. Int. Ed..

[24] Kalb R.S., Stepurko E.N., Emel’yanenko V.N., Verevkin S.P. (2016). Carbonate based ionic liquid synthesis (CBILS®): Thermodynamic analysis. Phys. Chem. Chem. Phys..

[25] Klauke K., Zaitsau D.H., Buelow M., He L., Klopotowski M., Knedel T.-O., Barthel J., Held C., Verevkin S.P., Janiak C. (2018). Thermodynamic properties of selenoether-functionalized ionic liquids and their use for the synthesis of zinc selenide nanoparticles. Dalton Trans..

[26] Zaitsau D.H., Verevkin S.P. (2018). Imidazolium-Based Ionic Liquids Containing the Trifluoroacetate Anion: Thermodynamic Study. J. Sol. Chem..

[27] Zaitsau D.H., Yermalayeu A.V., Pimerzin A.A., Verevkin S.P. (2018). Thermodynamics of Imidazolium-Based Ionic Liquids Containing the Trifluoromethanesulfonate Anion. Chem. Eng. Technol..

[28] Zaitsau D.H., Yermalayeu A.V., Pimerzin A.A., Verevkin S.P. (2018). Imidazolium based ionic liquids containing methanesulfonate anion: Comprehensive thermodynamic study. Chem. Eng. Res. Des..

[29] Paduszyński K. (2017). An overview of the performance of the COSMO-RS approach in predicting the activity coefficients of molecular solutes in ionic liquids and derived properties at infinite dilution. Phys. Chem. Chem. Phys..

[30] Cruickshank A.J.B., Windsor M.L., Young C.L. (1966). The use of gas-liquid chromatography to determine activity coefficients and second virial coefficients of mixtures I. Theory and verification of method of data analysis. Proc. R. Soc..

[31] Reid R.C., Prausnitz J.M., Sherwood T.K. (1977). The Properties of Gases and Liquids.

[32] Grant D.W. (1971). Gas-Liquid Chromatography.

[33] Flory P. (1953). Principles of Polymer Chemistry.

[34] Yoo B., Afzal W., Prausnitz J.M. (2012). Solubility Parameters for Nine Ionic Liquids. Ind. Eng. Chem. Res..

[35] Hansen C.M. (2004). 50 Years with solubility parameters—Past and future. Prog. Org. Coatings.

[36] Majer V., Svoboda V. (1985). Enthalpies of Vaporization of Organic Compounds: A Critical Review and Data Compilation.

[37] Lide D.R. (2009). CRC Handbook of Chemistry and Physics: A Ready-Reference Book of Chemical and Physical Data.

[38] Ionic Liquids Database—ILThermo (v2.0). https://ilthermo.boulder.nist.gov/.

[39] Domanska U., Marciniak A., Krolikowska M., Arasimowicz M. (2010). Activity Coefficients at Infinite Dilution Measurements for Organic Solutes and Water in the Ionic Liquid 1-Hexyl-3-methylimidazolium Thiocyanate. J. Chem. Eng. Data.

[40] Verevkin S.P., Zaitsau D.H., Tong B., Welz-Biermann U. (2011). New for old. Password to the thermodynamics of the protic ionic liquids. Phys. Chem. Chem. Phys..

[41] Khachatrian A.A., Shamsutdinova Z.I., Rakipov I.T., Varfolomeev M.A., Solomonov B.N., Verevkin S.P. (2018). Hydrogen bonding of molecular solutes in protic and aprotic ionic liquids. J. Mol. Liq..

[42] Reichardt C. (2005). Polarity of ionic liquids determined empirically by means of solvatochromic pyridinium N-phenolate betaine dyes. Green Chem..

[43] Jiang L.-K., Wang L.-S., Du C.-J., Wang X.-Y. (2014). Activity coefficients at infinite dilution of organic solutes in 1-hexyl-3-methylimidazolium trifluoroacetate and influence of interfacial adsorption using gas–liquid chromatography. J. Chem. Thermodyn..

[44] Marsh K.N., Brennecke J.F., Chirico R.D., Frenkel M., Heintz A., Magee J.W., Peters C.J., Rebelo L.P.N., Seddon K.R. (2009). Thermodynamic and thermophysical properties of the reference ionic liquid: 1-Hexyl-3-methylimidazolium bis[(trifluoromethyl)sulfonyl]amide (including mixtures). Part 1. Experimental methods and results (IUPAC Technical Report). Pure Appl. Chem..

[45] Heintz A., Verevkin S.P., Ondo D. (2006). Thermodynamic Properties of Mixtures Containing Ionic Liquids. 8. Activity Coefficients at Infinite Dilution of Hydrocarbons, Alcohols, Esters, and Aldehydes in 1-Hexyl-3-methylimidazolium Bis(trifluoromethylsulfonyl) Imide Using Gas−Liquid Chromatography. J. Chem. Eng. Data.

[46] Kato R., Gmehling J. (2005). Systems with ionic liquids: Measurement of VLE and γ∞ data and prediction of their thermodynamic behavior using original UNIFAC, mod. UNIFAC(Do) and COSMO-RS(Ol). J. Chem. Thermodyn..

[47] Letcher T.M., Marciniak A., Marciniak M., Domanska U. (2005). Activity coefficients at infinite dilution measurements for organic solutes in the ionic liquid 1-hexyl-3-methyl-imidazolium bis(trifluoromethylsulfonyl)-imide using g.l.c. at T = (298.15, 313.15, and 333.15) K. J. Chem. Thermodyn..

[48] Dobryakov Y.G., Tuma D., Maurer G. (2008). Activity Coefficients at Infinite Dilution of Alkanols in the Ionic Liquids 1-Butyl-3-methylimidazolium Hexafluorophosphate, 1-Butyl-3-methylimidazolium Methyl Sulfate, and 1-Hexyl-3-methylimidazolium Bis(trifluoromethylsulfonyl) Amide Using the Dilutor Technique. J. Chem. Eng. Data.

[49] Heintz A., Kulikov D.V., Verevkin S.P. (2002). Thermodynamic Properties of Mixtures Containing Ionic Liquids. 2. Activity Coefficients at Infinite Dilution of Hydrocarbons and Polar Solutes in 1-Methyl-3-ethyl-imidazolium Bis(trifluoromethyl-sulfonyl) Amide and in 1,2-Dimethyl-3-ethyl-imidazolium Bis(trifluoromethyl-sulfonyl) Amide Using Gas−Liquid Chromatography. J. Chem. Eng. Data.

[50] Krummen M., Wasserscheid P., Gmehling J. (2002). Measurement of Activity Coefficients at Infinite Dilution in Ionic Liquids Using the Dilutor Technique. J. Chem. Eng. Data.

[51] Deenadayalu N., Letcher T.M., Reddy P. (2005). Determination of Activity Coefficients at Infinite Dilution of Polar and Nonpolar Solutes in the Ionic Liquid 1-Ethyl-3-methyl- imidazolium Bis(trifluoromethylsulfonyl) Imidate Using Gas−Liquid Chromatography at the Temperature 303.15 K or 318.15 K. J. Chem. Eng. Data.

[52] Heintz A., Casás L.M., Nesterov I.A., Emel’yanenko V.N., Verevkin S.P. (2005). Thermodynamic Properties of Mixtures Containing Ionic Liquids. 5. Activity Coefficients at Infinite Dilution of Hydrocarbons, Alcohols, Esters, and Aldehydes in 1-Methyl-3-butyl-imidazolium Bis(trifluoromethyl-sulfonyl) Imide Using Gas−Liquid Chromatography. J. Chem. Eng. Data.

[53] Singh S., Bahadur I., Naidoo P., Redhi G., Ramjugernath D. (2016). Application of 1-butyl-3-methylimidazolium bis(trifluoromethylsulfonyl) imide ionic liquid for the different types of separations problem: Activity coefficients at infinite dilution measurements using gas-liquid chromatography technique. J. Mol. Liq..

[54] Zhang T., Bao Y.-N., Zhang L., Ren R.-Z., Jiao Y.-H., Ge M.-L. (2020). Thermodynamics and selectivity of separation based on activity coefficients at infinite dilution of various solutes in ionic liquid [DMIM][Tf2N]. J. Chem. Thermodyn..

[55] Domanska U., Wlazło M. (2016). Thermodynamics and limiting activity coefficients measurements for organic solutes and water in the ionic liquid 1-dodecyl-3-methylimidzolium bis(trifluoromethylsulfonyl) imide. J. Chem. Thermodyn..

[56] Domanska U., Marciniak A. (2007). Activity Coefficients at Infinite Dilution Measurements for Organic Solutes and Water in the Ionic Liquid 1-Ethyl-3-methylimidazolium Trifluoroacetate. J. Phys. Chem. B.

[57] Olivier E., Letcher T.M., Naidoo P., Ramjugernath D. (2010). Activity coefficients at infinite dilution of organic solutes in the ionic liquid 1-ethyl-3-methylimidazolium trifluoromethanesulfonate using gas–liquid chromatography at T = (313.15, 323.15, and 333.15) K. J. Chem. Thermodyn..

[58] Ge M.-L., Wang L.-S., Li M.-Y., Wu J.-S. (2007). Activity Coefficients at Infinite Dilution of Alkanes, Alkenes, and Alkyl Benzenes in 1-Butyl-3-methylimidazolium Trifluoromethanesulfonate Using Gas−Liquid Chromatography. J. Chem. Eng. Data.

[59] Ge M.-L., Wang L.-S. (2008). Activity Coefficients at Infinite Dilution of Polar Solutes in 1-Butyl-3-methylimidazolium Trifluoromethanesulfonate Using Gas–Liquid Chromatography. J. Chem. Eng. Data.

[60] Domanska U., Marciniak A. (2008). Activity Coefficients at Infinite Dilution Measurements for Organic Solutes and Water in the Ionic Liquid 1-Butyl-3-methylimidazolium Trifluoromethanesulfonate. J. Phys. Chem. B.

[61] Yang X.-J., Wu J.-S., Ge M.-L., Wang L.-S., Li M.-Y. (2008). Activity Coefficients at Infinite Dilution of Alkanes, Alkenes, and Alkyl Benzenes in 1-Hexyl-3-methylimidazolium Trifluoromethanesulfonate Using Gas−Liquid Chromatography. J. Chem. Eng. Data.

[62] Moïse J.-C., Mutelet F., Jaubert J.-N., Grubbs L.M., Acree W.E., Baker G.A. (2011). Activity Coefficients at Infinite Dilution of Organic Compounds in Four New Imidazolium-Based Ionic Liquids. J. Chem. Eng. Data.

[63] Domanska U., Królikowski M. (2012). Measurements of activity coefficients at infinite dilution for organic solutes and water in the ionic liquid 1-ethyl-3-methylimidazolium methanesulfonate. J. Chem. Thermodyn..

[64] Blahut A., Sobota M., Dohnal V., Vrbka P. (2010). Activity coefficients at infinite dilution of organic solutes in the ionic liquid 1-ethyl-3-methylimidazolium methanesulfonate. Fluid Phase Equilib..

[65] Martins M.A., Coutinho J.A., Pinho S.P., Domańska U. (2015). Measurements of activity coefficients at infinite dilution of organic solutes and water on polar imidazolium-based ionic liquids. J. Chem. Thermodyn..

[66] Zaitsau D.H., Siewert R., Pimerzin A.A., Bülow M., Held C., Loor M., Schulz S., Verevkin S.P. (2021). From volatility to solubility: Thermodynamics of imidazolium-based ionic liquids containing chloride and bromide anions. J. Mol. Liq..

[67] Zhang M., He Z.-Z., Kang R.-X., Ge M.-L. (2019). Thermodynamics and activity coefficients at infinite dilution for organic compounds in the ionic liquid 1-hexyl-3-methylimidazolium chloride. J. Chem. Thermodyn..

[68] Foco G.M., Bottini S.B., Quezada N., de la Fuente J.C., Peters C.J. (2006). Activity Coefficients at Infinite Dilution in 1-Alkyl-3-methylimidazolium Tetrafluoroborate Ionic Liquids. J. Chem. Eng. Data.

[69] Ge M.-L., Wang L.-S., Wu J.-S., Zhou Q. (2008). Activity Coefficients at Infinite Dilution of Organic Solutes in 1-Ethyl-3-methylimidazolium Tetrafluoroborate Using Gas−Liquid Chromatography. J. Chem. Eng. Data.

[70] Zhang J., Zhang Q., Qiao B., Deng Y. (2007). Solubilities of the Gaseous and Liquid Solutes and Their Thermodynamics of Solubilization in the Novel Room-Temperature Ionic Liquids at Infinite Dilution by Gas Chromatography. J. Chem. Eng. Data.

[71] Revelli A.-L., Mutelet F., Turmine M., Solimando R., Jaubert J.-N. (2009). Activity Coefficients at Infinite Dilution of Organic Compounds in 1-Butyl-3-methylimidazolium Tetrafluoroborate Using Inverse Gas Chromatography. J. Chem. Eng. Data.

[72] Zhou Q., Wang L.-S., Wu J.-S., Li M.-Y. (2007). Activity Coefficients at Infinite Dilution of Polar Solutes in 1-Butyl-3-methylimidazolium Tetrafluoroborate Using Gas−Liquid Chromatography. J. Chem. Eng. Data.

[73] Zhou Q., Wang L.-S. (2006). Activity Coefficients at Infinite Dilution of Alkanes, Alkenes, and Alkyl Benzenes in 1-Butyl-3-methylimidazolium Tetrafluoroborate Using Gas−Liquid Chromatography. J. Chem. Eng. Data.

[74] Sudhir N., Yadav P., Sah R., Nautiyal B., Ghosh P., Nanoti S.M., Singh R. (2019). Measuring Activity Coefficient at Infinite Dilution of Hydrocarbons in Ionic Liquids and Evaluation of Other Thermodynamic Properties using Gas Chromatography. J. Chem. Eng. Data.

[75] Bahlmann M., Nebig S., Gmehling J. (2009). Activity coefficients at infinite dilution of alkanes and alkenes in 1-alkyl-3-methylimidazolium tetrafluoroborate. Fluid Phase Equilib..

[76] Letcher T.M., Soko B., Reddy P., Deenadayalu N. (2003). Determination of Activity Coefficients at Infinite Dilution of Solutes in the Ionic Liquid 1-Hexyl-3-methylimidazolium Tetrafluoroborate Using Gas−Liquid Chromatography at the Temperatures 298.15 K and 323.15 K. J. Chem. Eng. Data.

[77] Heintz A., Verevkin S.P. (2005). Thermodynamic Properties of Mixtures Containing Ionic Liquids. 6. Activity Coefficients at Infinite Dilution of Hydrocarbons, Alcohols, Esters, and Aldehydes in 1-Methyl-3-octyl-imidazolium Tetrafluoroborate Using Gas−Liquid Chromatography. J. Chem. Eng. Data.

[78] Li Y., Wang L.-S., Li M.-Y., Tian N.-N. (2011). Activity Coefficients at Infinite Dilution of Organic Solutes in 1-Decyl-3-methylimidazolium Tetrafluoroborate Using Gas−Liquid Chromatography. J. Chem. Eng. Data.

[79] Martins M.A.R., Vilas-Boas S.M., Cordova I.W., Carvalho P.J., Domańska U., Ferreira O., Coutinho J.A.P., Pinho S.P. (2021). Infinite Dilution Activity Coefficients in the Smectic and Isotropic Phases of Tetrafluoroborate-Based Ionic Liquids. J. Chem. Eng. Data.

[80] Mutelet F., Jaubert J.-N. (2007). Measurement of activity coefficients at infinite dilution in 1-hexadecyl-3-methylimidazolium tetrafluoroborate ionic liquid. J. Chem. Thermodyn..

[81] Mutelet F., Butet V., Jaubert J.-N. (2005). Application of Inverse Gas Chromatography and Regular Solution Theory for Characterization of Ionic Liquids. Ind. Eng. Chem. Res..

[82] Shimoyama Y., Hirayama T., Iwai Y. (2008). Measurement of Infinite Dilution Activity Coefficients of Alcohols, Ketones, and Aromatic Hydrocarbons in 4-Methyl-N-butylpyridinium Tetrafluoroborate and 1-Butyl-3-methylimidazolium Hexafluorophosphate by Gas−Liquid Chromatography. J. Chem. Eng. Data.

[83] Zhu J., Yu Y., Chen J., Fei W. (2007). Measurement of activity coefficients at infinite dilution for hydrocarbons in imidazolium-based ionic liquids and QSPR model. Model Front. Chem. Eng. China.

[84] Xu Q., Su B., Luo X., Xing H., Bao Z., Yang Q., Yang Y., Ren Q. (2012). Accurate Measurements of Infinite Dilution Activity Coefficients Using Gas Chromatography with Static-Wall-Coated Open-Tubular Columns. Anal. Chem..

[85] Letcher T.M., Soko B., Ramjugernath D., Deenadayalu N., Nevines A., Naicker P.K. (2003). Activity Coefficients at Infinite Dilution of Organic Solutes in 1-Hexyl-3-methylimidazolium Hexafluorophosphate from Gas−Liquid Chromatography. J. Chem. Eng. Data.

[86] Li Y., Wang L.-S., Feng Y.-X., Zhang C.-Y. (2011). Activity Coefficients of Organic Solutes at Infinite Dilution in Ionic Liquids. 1. 1-Hexyl-3-Methylimidazolium Hexafluorophosphate and 1-Octyl-3-Methylimidazolium Hexafluorophosphate and Their Application to Alkane/Aromatic and Aromatic/Aromatic Hydrocarbon Separation. Ind. Eng. Chem. Res..

[87] Deng L., Wang Q., Chen Y., Zhang Z., Tang J. (2013). Determination of the solubility parameter of ionic liquid 1-octyl-3-methylimidazolium hexafluorophosphate by inverse gas chromatography. J. Mol. Liq..

[88] Olivier E., Letcher T.M., Naidoo P., Ramjugernath D. (2010). Activity coefficients at infinite dilution of organic solutes in the ionic liquid 1-octyl-3-methylimidazolium hexafluorophosphate using gas–liquid chromatography at T = (313.15, 323.15, and 333.15) K. J. Chem. Thermodyn..

[89] Zaitsau D.H., Verevkin S.P. (2019). Imidazolium-based ionic liquids containing FAP anion: Thermodynamic study. J. Mol. Liq..

[90] Wlazło M., Marciniak A., Letcher T.M. (2015). Activity Coefficients at Infinite Dilution and Physicochemical Properties for Organic Solutes and Water in the Ionic Liquid 1-Ethyl-3-methylimidazolium trifluorotris(perfluoroethyl)phosphate. J. Sol. Chem..

[91] Yan P.-F., Yang M., Liu X.-M., Liu Q.-S., Tan Z.-C., Welz-Biermann U. (2010). Activity Coefficients at Infinite Dilution of Organic Solutes in 1-Ethyl-3-methylimidazolium Tris(pentafluoroethyl)trifluorophosphate [EMIM][FAP] Using Gas−Liquid Chromatography. J. Chem. Eng. Data.

[92] Emel’yanenko V.N., Verevkin S.P., Heintz A., Schick C. (2008). Ionic Liquids. Combination of Combustion Calorimetry with High-Level Quantum Chemical Calculations for Deriving Vaporization Enthalpies. J. Phys. Chem. B.

[93] Sobota M., Dohnal V., Vrbka P. (2009). Activity Coefficients at Infinite Dilution of Organic Solutes in the Ionic Liquid 1-Ethyl-3-methyl-imidazolium Nitrate. J. Phys. Chem. B.

[94] Foco G., Bermejo M.D., Kotlewska A.J., van Rantwijk F., Peters C.J., Bottini S.B. (2011). Activity Coefficients at Infinite Dilution in Methylimidazolium Nitrate Ionic Liquids. J. Chem. Eng. Data.

[95] Feng Y.-X., Wang L.-S., Li Y. (2011). Activity Coefficients at Infinite Dilution of Organic Solutes in 1-Butyl-3-methylimidazolium Nitrate Using Gas−Liquid Chromatography. J. Chem. Eng. Data.

[96] Kan J., Wang L.-S., Wang X.-X., Duan J.-D. (2012). Activity Coefficients of Organic Solutes at Infinite Dilution in the Ionic Liquids. 2. Organic Solutes in 1-Hexyl-3-methylimidazolium Nitrate and Gas–Liquid Partitioning and Interfacial Adsorption Using Gas–Liquid Chromatography. Ind. Eng. Chem. Res..

[97] Duan J.-D., Wang L.-S., Jiang K., Wang X.-X. (2012). Activity coefficients at infinite dilution of organic solutes in 1-octyl-3-methylimidazolium nitrate using gas–liquid chromatography. Fluid Phase Equilib..

[98] Zaitsau D.H., Emel’yanenko V.N., Verevkin S.P., Heintz A. (2010). Sulfur-Containing Ionic Liquids. Rotating-Bomb Combustion Calorimetry and First-Principles Calculations for 1-Ethyl-3-methylimidazolium Thiocyanate. J. Chem. Eng. Data.

[99] Deyko A., Lovelock K.R.J., Corfield J.-A., Taylor A.W., Gooden P.N., Villar-Garcia I.J., Licence P., Jones R.G., Krasovskiy V.G., Chernikova E.A. (2009). Measuring and predicting Δ_vap_H_298_ values of ionic liquids. Phys. Chem. Chem. Phys..

[100] Domańska U., Marciniak A. (2008). Measurements of activity coefficients at infinite dilution of aromatic and aliphatic hydrocarbons, alcohols, and water in the new ionic liquid [EMIM][SCN] using GLC. J. Chem. Thermodyn..

[101] Domanska U., Laskowska M. (2009). Measurements of activity coefficients at infinite dilution of aliphatic and aromatic hydrocarbons, alcohols, thiophene, tetrahydrofuran, MTBE, and water in ionic liquid [BMIM][SCN] using GLC. J. Chem. Thermodyn..

[102] Mutelet F., Revelli A.-L., Jaubert J.-N., Sprunger L.M., Acree W.E., Baker G.A. (2010). Partition Coefficients of Organic Compounds in New Imidazolium and Tetralkylammonium Based Ionic Liquids Using Inverse Gas Chromatography. J. Chem. Eng. Data.

[103] Domanska U., Wlazło M., Karpinska M. (2016). Activity coefficients at infinite dilution of organic solvents and water in 1-butyl-3-methylimidazolium dicyanamide. A literature review of hexane/hex-1-ene separation. Fluid Phase Equilib..

[104] Emel’yanenko V.N., Zaitsau D.H., Verevkin S.P., Heintz A., Voß K., Schulz A. (2011). Vaporization and Formation Enthalpies of 1-Alkyl-3-methylimidazolium Tricyanomethanides. J. Phys. Chem. B.

[105] Karpinska M., Wlazło M., Domanska U. (2017). Separation of binary mixtures based on gamma infinity data using [EMIM][TCM] ionic liquid and modelling of thermodynamic functions. J. Mol. Liq..

[106] Lukoshko E., Mutelet F., Domanska U. (2015). Experimental and theoretically study of interaction between organic compounds and tricyanomethanide based ionic liquids. J. Chem. Thermodyn..

[107] Zaitsau D.H., Pohako-Esko K., Arlt S., Emel’yanenko V.N., Schulz P.S., Wasserscheid P., Schulz A., Verevkin S.P. (2017). Thermodynamics of imidazolium based ionic liquids with cyano containing anions. J. Mol. Liq..

[108] Domańska U., Królikowska M., Acree W.E., Baker G.A. (2011). Activity coefficients at infinite dilution measurements for organic solutes and water in the ionic liquid 1-ethyl-3-methylimidazolium tetracyanoborate. J. Chem. Thermodyn..

[109] Yan P.-F., Yang M., Liu X.-M., Wang C., Tan Z.-C., Welz-Biermann U. (2010). Activity coefficients at infinite dilution of organic solutes in the ionic liquid 1-ethyl-3-methylimidazolium tetracyanoborate [EMIM][TCB] using gas–liquid chromatography. J. Chem. Thermodyn..

[110] Domańska U., Lukoshko E.V., Wlazło M. (2012). Measurements of activity coefficients at infinite dilution for organic solutes and water in the ionic liquid 1-hexyl-3-methylimidazolium tetracyanoborate. J. Chem. Thermodyn..

[111] Domańska U., Marciniak A. (2010). Physicochemical Properties and Activity Coefficients at Infinite Dilution for Organic Solutes and Water in the Ionic Liquid 1-Decyl-3-methylimidazolium Tetracyanoborate. J. Phys. Chem. B.

