# Supplementary material for: Imidazolium Based Ionic Liquids: Unbiased Recovering of Vaporization Enthalpies from Infinite-Dilution Activity Coefficients
Source: Molecules. 2021 Sep 28;26(19):5873. doi: 10.3390/molecules26195873 (PMC8510351; doi:10.3390/molecules26195873)
Supplement: Supplementary file 1 [file molecules-26-05873-s001.zip › molecules-1383594-supplementary.pdf]

Molecules, 2021 (Electronic Supporting Information)

Imidazolium based Ionic Liquids: unbiased recovering of vaporization enthalpies from infinite-dilution activity coefficients

Sergey P. Verevkin[a,b]

[a]*Department of Physical Chemistry and Department of „Science and Technology of Life, Light and Matter“, University of Rostock, 18059, Dr-Lorenz-Weg 2, Rostock, Germany*
[b] *Competence Centre CALOR, Faculty of Interdisciplinary Research, University of Rostock, 18059 Rostock, Germany*

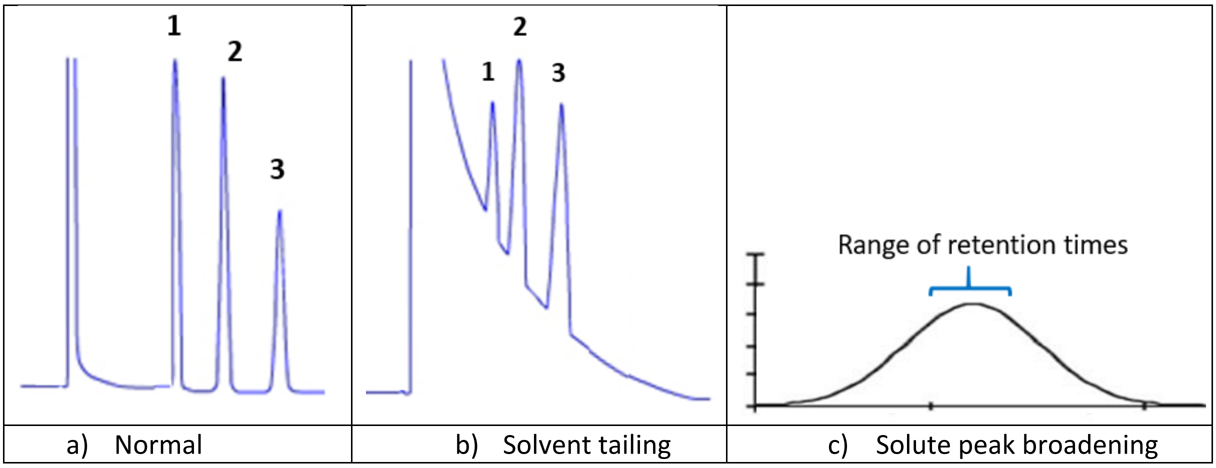

Fig. S1. Typical gas-chromatographic charts from γ_1^∞-measurements.
1: n-pentane; 2: n-hexane, 3: n-heptane

Table S1. Data from Ref. [1] used for regression with Eq. (4 and 5) for [C_6mim][SCN]: solubility parameters $\delta_{298.15\ K}$ of different solutes in at 298.15 K and the left part of Eq. (5)

solute	γ_1^{∞}(298.15 K)	$\delta_{298.15\ K}$ /MPa$^{0.5}$	Y[a]
Heptane	81.4	15.2	0.0583
Octane	95.7	15.4	0.0648
Nonane	120	15.7	0.0705
Decane	162	15.8	0.0729
Cyclopentane	23.2	16.6	0.0739
Cyclohexane	28.2	16.8	0.0809
Cycloheptane	25.8	17.2	0.0934
Cyclooctane	30.1	17.4	0.0989
Hex-1-ene	28.7	14.9	0.0576
Hept-1-ene	34.2	15.3	0.0658
Oct-1-ene	42.1	15.5	0.0710
Pent-1-yne	4.04	16.2	0.0871
Hex-1-yne	4.97	16.4	0.0924
Hept-1-yne	6.69	16.4	0.0929
Oct-1-yne	9.15	16.4	0.0929
Benzene	1.91	18.8	0.1339
Toluene	2.79	18.2	0.1248
Ethylbenzene	4.32	18.0	0.1194
o-Xylene	3.53	18.4	0.1268
m-Xylene	4.37	18.0	0.1202
p-Xylene	4.18	17.9	0.1192
Methanol	0.532	29.4	0.3450
Ethanol	0.844	26.1	0.2700
Propan-1-ol	0.990	24.5	0.2376
Butan-1-ol	1.25	23.3	0.2155
Acetone	1.25	19.6	0.1480
Thiophene	1.24	20.2	0.1615
THF	1.60	19.0	0.1386
MTBE	8.78	15.1	0.0717
di-Et ether	8.67	15.4	0.0707
di-Pr ether	18.9	15.6	0.0750
di-Bu ether	33.9	15.8	0.0801
Water	0.59	47.9	0.8953

[a]The left part of Eq. (5): $Y = (\delta_1)^2/(RT) - \chi_{12}/V_1^*$

Table S2 Data from Ref. [2] used for regression with Eq. (4 and 5) for $[C_6mim][CF_3CO_2]$: solubility parameters $\delta_{298.15\ K}$ of different solutes in at 298.15 K and the left part of Eq. (5)

solute	$\delta_{298.15\ K}$ /MPa$^{0.5}$	Y[a]
benzene	18.8	0.1325
toluene	18.2	0.1238
ethylbenzene	18.0	0.1194
o-xylene	18.4	0.1266
m-xylene	18.0	0.1202
p-xylene	17.9	0.119
1-propanol	24.5	0.2563
2-propanol	23.6	0.2345
2-methyl-1-propanol	22.9	0.2219
acetonitrile	24.1	0.2230
ethyl acetate	18.4	0.1267
acetone	19.6	0.1426
tetrhydrofuran	19.0	0.1357
1.4-dioxan	20.6	0.1639
dichloromethane	20.2	0.1679
trichloromethane	18.9	0.1631
1.2-dichloromethane	20.3	0.1703
chlorobenzene	19.5	0.1467
bromobenzene	19.9	0.1558
styrene	18.8	0.1366

[a]The left part of Eq. (5): $Y = (\delta_1)^2/(RT) - \chi_{12}/V_1^*$

Table S3 [$C_n$mim][NTf_2] series: comparison of $\Delta_l^g H_m^o$(298.15 K)-values derived according to Eqs. 5 and 6 with experimental results from conventional methods

IL	$\Delta_l^g H_m^o$(exp)[a]	$\Delta_l^g H_m^o$(GLC)[b]	$\Delta_l^g H_m^o$(*HE*)[c]	F_{im} [d]	Ref. [e]
[C_2mim][NTf_2]	127.7±1.8 [10]	119.0	121.3	1.05	49
		115.3	119.3	1.07	50
		133.3			51
[C_4mim][NTf_2]	134.9±1.7 [10]	129.2	130.1	1.04	52
			128.8	1.05	53
[C_6mim][NTf_2]	139.9±1.8 [10]	133.9	133.9	1.04	45
		131.4	135.5	1.03	46
		143.5			47
			138.0 [f]	1.01	45,48
[C_8mim][NTf_2]	147.1±2.0 [10]	145.8	143.8	1.02	46
[C_{10}mim][NTf_2]	154.6±2.5 [10]	151.1	152.5	1.01	54
[C_{12}mim][NTf_2]	161.5±1.8 [10]		157.0	1.03	55
				1.04±0.01	

[a] Experimental values derived from different conventional techniques and evaluated and reported in our recent papers.
[b] Estimated according to Eqs. 5 and 6 using γ_1^∞-values for all solutes reported in original papers.
[c] Estimated according to Eqs. 5 and 6 using γ_1^∞-values only for n-alkanes and alcohols reported in original papers.
[d] Correction factor $F_{im} = \Delta_l^g H_m^o(\mathrm{exp}) / \Delta_l^g H_m^o(HM)$.
[e] References for the experimental γ_1^∞-values are the same as in the main text.
[f] Combination of γ_1^∞-data set of alcohols [48] with the γ_1^∞-data set for n-alkanes [45].

Table S4 [$C_n$mim][CF_3CO_2] series: comparison of $\Delta_l^g H_m^o$(298.15 K)-values derived according to Eqs. 5 and 6 with experimental results from conventional methods [a]

IL	$\Delta_l^g H_m^o$(exp)	$\Delta_l^g H_m^o$(GLC)	$\Delta_l^g H_m^o$(*HE*)	F_{im}	Ref.
[C_2mim][CF_3CO_2]	126.4±1.5 [26]	122.3	118.5	1.07	56
[C_6mim][CF_3CO_2]	141.5±1.9 [26]	183.2			43
				1.07±0.09	

[a] The definition of symbols is the same as in Table S3.

Table S5 [$C_n$mim][Anion] series with [CF_3SO_3], [CH_3SO_3], and [Cl]: comparison of $\Delta_l^g H_m^o$(298.15 K)-values derived according to Eqs. 5 and 6 with experimental results from conventional methods[a]

IL	$\Delta_l^g H_m^o$(exp)	$\Delta_l^g H_m^o$(GLC)	$\Delta_l^g H_m^o$(HE)	F_{im}	Ref.
[C_2mim][CF_3SO_3]	134.2±2.5[27]	108.0	106.5	1.26	57
[C_4mim][CF_3SO_3]	138.7±2.4[27]	116.7[b]	114.9	1.21	58,59
		121.2	118.6	1.17	60
[C_6mim][CF_3SO_3]	146.1±2.4[27]	137.6[c]			61
				1.21±0.03	
[C_2mim][CH_3SO_3]	141.4±1.9[28]	120.5	112.2	1.26	62
		111.8	112.9	1.25	63
		122.7	114.7	1.23	64
[C_4mim][CH_3SO_3]	149.8±2.1[28]	126.5	128.9	1.16	65
				1.23±0.02	
[C_4mim][Cl]	153.3±1.9[66]	140.5	128.9	1.19	65
[C_6mim][Cl]	160.5±2.4[66]	132.2	125.7	1.28	67
[C_8mim][Cl]	166.8±3.8[66]		139.7	1.19	65
				1.22±0.03	

[a] The definition of symbols is the same as in Table S3.

[b] Combination of γ_1^∞-data set of n-alkanes [58] with the γ_1^∞-data set for alcohols [59].

[c] Estimated from data set containing γ_1^∞-values only for alkylbenzenes and low polar solutes

Table S6 [$C_n$mim][Anion] series with fluorine-containing anions: comparison of $\Delta_l^g H_m^o$(298.15 K)-values derived according to Eqs. 5 and 6 with experimental results from conventional methods [a]

IL	$\Delta_l^g H_m^o$(exp)	$\Delta_l^g H_m^o$(GLC)	$\Delta_l^g H_m^o$(HE)	F_{im}	Ref.
[C_2mim][BF_4]	130.5±2.9[16]	101.1	98.5	1.32	68
		95.3			69
[C_4mim][BF_4]	138.3±2.7[16]	106.5	103.8	1.33	70
		105.0	102.5	1.35	71
		96.4	95.8	1.44	68
		99.3	95.6 [c]	1.45	72,73
			92.1 [c]	1.50	71,74
			97.6 [c]	1.42	71,75
[C_6mim][BF_4]	146.8±2.7[16]	118.4			76
		107.9	107.5	1.37	68
			107.9 [c]	1.36	68,75
[C_8mim][BF_4]	152.8±2.8[16]	117.0	114.8	1.33	77
		116.7	114.6	1.33	68
		142.9	131.4	1.16	70
[C_{10}mim][BF_4]	158.3±2.8[16]	132.4			78
[C_{12}mim][BF_4]	167.3±2.9		126.4	1.32 [e]	79
			124.7	1.34 [f]	79
[C_{14}mim][BF_4]	179.4±3.1		141.4	1.27 [e]	79
			131.1	1.37 [f]	79
[C_{16}mim][BF_4]	180.5±3.5 [d]	143.6	136.7	1.32	80
				1.35±0.15	
[C_4mim][PF_6]	146.5±2.6[13]	96.0	96.1	1.52	81
		75.4	93.3 [c]	1.57	81,82
		124.5	94.0 [c]	1.56	81,83
			96.8	1.51	84
			93.4 [c]	1.57	48,81
[C_6mim][PF_6]	150.8±2.7[13]	111.0			85
		85.7	92.3	1.63	86
[C_8mim][PF_6]	154.9±2.8[13]	128.7 [c]			86,87
		128.5	123.6	1.25	87
		115.0	110.9	1.40	88
				1.50±0.04	
[C_2mim][FAP] [g]	126.4±1.9[89]	130.1	133.8	0.94	90
		123.3	127.0	1.00	91
[C_6mim][FAP] [g]	141.5±1.9[89]	158.0	148.9	0.95	62
				0.96±0.02	

[a] The definition of symbols is the same as in Table S3.
[b] Experimental data on alcohols were absent in this data set.
[c] Combination of γ_1^∞-data set of alcohols with the γ_1^∞-data set for n-alkanes reported in different papers, as given in the last column.
[d] Extrapolated value based on experimental data [16].
[e] Activity coefficients were measure in the liquid phase.
[f] Activity coefficients were measure in the liquid-crystal phase.
[g] [FAP] = tris(pentafluoroethyl)trifluorophosphate anion.

Table S7 [$C_n$mim][NO_3] series: comparison of $\Delta_l^g H_m^o$(298.15 K)-values derived according to Eqs. 5 and 6 with experimental results from conventional methods [a]

IL	$\Delta_l^g H_m^o$(exp)	$\Delta_l^g H_m^o$(GLC)	$\Delta_l^g H_m^o$(HE)	F_{im}	Ref.
[C_2mim][NO_3]	158.1±5.3[92]	90.0	88.0	1.80	93
[C_4mim][NO_3]	162.3±5.3[92]	109.5	108.3	1.50	94
[C_4mim][NO_3]	162.3±5.3[92]	112.3	106.1	1.53	95
[C_6mim][NO_3]	170.1±5.3[92]	121.6 [b]	108.8	1.56	96
[C_8mim][NO_3]	177.9±5.3[92]	136.5	131.3	1.35	97
				1.55±0.07	

[a] The definition of symbols is the same as in Table S3.
[b] The γ_1^∞-data set for n-alkanes were not reported in the original work. They were assessed in this work according to the trend with the chain-length.

Table S8 [$C_n$mim][Anion] series with cyano-containig anions: comparison of $\Delta_l^g H_m^o$(298.15 K)-values derived according to Eqs. 5 and 6 with experimental results from conventional methods [a]

IL	$\Delta_l^g H_m^o$(exp)	$\Delta_l^g H_m^o$(GLC)	$\Delta_l^g H_m^o$(HE)	F_{im}	Ref.
[C_2mim][SCN]	153.6±1.8[104,105]	100.9	96.3	1.60	98
[C_4mim][SCN]	156.9±2.5[104]	116.4	113.9	1.38	99
[C_6mim][SCN]	164.7±3.0[104]	122.8	118.9	1.39	39
				1.46±0.07	
[C_2mim][DCA]	155.6±3.1[3,5]	102.8	100.3	1.55	100
[C_4mim][DCA]	162.9±3.4[3,5]	113.9	115.7	1.41	101
				1.48±0.07	
[C_2mim][TCM]	140.3±3.0[5,106]	100.8	101.4	1.38	102
[C_4mim][TCM]	146.9±3.0[5,106]	117.7	115.6	1.27	103
				1.33±0.06	
[C_2mim][TCB]	130.1±2.5[107]	98.1	96.7	1.35	108
		94.4	92.6	1.40	109
[C_6mim][TCB]	143.0±2.3[107]	120.8	119.5	1.20	110
[C_{10}mim][TCB]	157.1±2.5[107]	155.1	153.1	1.03	111
				1.24±0.08	

[a] The definition of symbols is the same as in Table S3.
[SCN] = thio-cyanate; [DCA] = di-cyano-amide; [TCM] = tri-cyano-methane, and [TCB] = tetra-cyano-borate.

References

1 U. Domanska, M. Krolikowska and M. Arasimowicz, M. Phase Equilibria of (1-Hexyl-3-methylimidazolium Thiocyanate + Water, Alcohol, or Hydrocarbon) Binary Systems. *J. Chem. Eng. Data*, 2010, **55**, 773−777.
2 L.-K. Jiang, L.-S. Wang, C.-J. Du and X.-Y Wang, Activity coefficients at infinite dilution of organic solutes in 1-hexyl-3-methylimidazolium trifluoroacetate and influence of interfacial adsorption using gas–liquid chromatography. *J. Chem. Thermodyn.*, 2014, **70**, 138–146.
